# Supplementary material for: LKB1 Loss Correlates with STING Loss and, in Cooperation with β-Catenin Membranous Loss, Indicates Poor Prognosis in Patients with Operable Non-Small Cell Lung Cancer
Source: Cancers (Basel). 2024 May 10;16(10):1818. doi: 10.3390/cancers16101818 (PMC11120022; doi:10.3390/cancers16101818)
Supplement: Supplementary file 1 [file cancers-16-01818-s001.zip › Supplementary material - Index review.pdf]

Supplementary material - Index

Supplementary material 1 (S1)

Table of antibodies and methodology

Supplementary material 2 (S2)

Assessment of immunohistochemical expression

Supplementary material 3 (S3)

Sequencies of the primers και Taqman probes

Supplementary material 4 (S4)

Table S4 - Overall - Clinicopathological Characteristics by Histotype

Supplementary material 5 (S5)

Table S5 - Overall - Laboratory Analysis by Histotype Laboratory Characteristics

Supplementary material 5 (S6)

Table S6 - Overall – KRAS and BRAF mutations

Supplementary material 7 (S7)

Table S7 - Non-pleomorphic LUACs vs pleomorphic LUACs – Laboratory Characteristics

Supplementary material 8 (S8)

Table S8 - Non-pleomorphic LSCCs vs pleomorphic LSCCs – Laboratory Characteristics

Supplementary material 9 (S9)

Table S9 - Overall - LKB1 loss vs LKB1 intact Clinicopathological Characteristics

Supplementary material 10 (S10)

Table S10 - LUACs - LKB1 loss vs LKB1 intact Laboratory Characteristics

Supplementary material 11 (S11)

Table S11 - LSCCs - LKB1 loss vs LKB1 intact _Clinicopathological Characteristics

Supplementary material 12 (S12)

Table S12 - LUACs & LKB1 loss vs LUACs & LKB1 intact _Clinical Variables

Supplementary material 13 (S13)

Table S13 - Overall LN+ - Metastatic tumors with LKB1 loss vs Metastatic tumors with LKB1 intact – Laboratory Variables

Supplementary material 14 (S14)

Table S14 - LUACs LN+ - Metastatic tumors with LKB1 loss vs Metastatic tumors with LKB1 intact – Laboratory Variables

Supplementary material 15 (S15)

Table S15 - LSCCs LN+ - Metastatic tumors with LKB1 loss vs Metastatic tumors with LKB1 intact – Laboratory Variables

Supplementary material 16 (S16)

Table S16 - Co-mutational Cohorts – Laboratory Variables

Supplementary material 17 (S17)

Table S17 - Co-mutational Cohorts – Clinical Variables

Supplementary material 18 (S18)

Table S18 - Overall – median Overall Survival

Supplementary material 19 (S19)

Table S19 - LKB1 loss _ median Overall Survival

Supplementary material 20 (S20)

Table S20 - KL-mOS_ median Overall Survival
