# Supplementary material for: LKB1 Loss Correlates with STING Loss and, in Cooperation with β-Catenin Membranous Loss, Indicates Poor Prognosis in Patients with Operable Non-Small Cell Lung Cancer
Source: Cancers (Basel). 2024 May 10;16(10):1818. doi: 10.3390/cancers16101818 (PMC11120022; doi:10.3390/cancers16101818)
Supplement: Supplementary file 1 [file cancers-16-01818-s001.zip › Supplementary material S1.pdf]

Supplementary material 1 (S1)

Table of antibodies and methodology

Antibody / Clone	Code	Source	Dilution in TBS / Incubation time	Antigen retrieval	Detection kit
LKB1 (Ley 37D/G6)	SC-32245	Santa Cruz Biotechnology	1:100 (60min*)	MW with EDTA	Ultravision Quanto Detection System HRP (TL-060-QHL) with DAB Quanto Brown HRP (TA-125-QHDX), Thermo Scientific.
p-AMPK (Thr172)(40H9)	2535	Cell Signaling Technology	1:150 (60min*)	MW with EDTA	
STING	D2P2F	Cell Signaling Technology	1:500 (60 min*)	MW with CB	
p16 (c-20)	SC-468	Santa Cruz Biotechnology	1:300 (Overnight at 4^0 C)	MW with EDTA	
p53 (DO-7)	M7001	Dako	1:600 (60 min*)	MW with EDTA	
Cyclin D1 (SP4)	RM-9104-S	Neomarkers	1:100 (Overnight at 4^0 C)	MW with EDTA	
CD24 (SN3b)	MS-1279-P	Neomarkers	1:200 (60 min*)	MW with EDTA	
VEGF-C	182255	Invitrogen	1:150 (Overnight at 4^0 C)	MW with EDTA	
PDGFR-α (C-20)	SC-338	Santa Cruz Biotechnology	1:100 (60 min*)	MW with EDTA	
PDGFR-β (P-20)	SC-339	Santa Cruz Biotechnology	1:200 (60 min*)	MW with EDTA	
ZEB-1	HPA 27524	Atlas Antibodies	1:800 (Overnight at 4^0 C)	MW with CB	

*At room temperature
MW = microwave heating (350 W, three times for 5 min. each)
EDTA = Ethylenediaminetetraacetic acid (0.001 M EDTA, pH 8)
CB = Citrate buffer (0.01 M Citrate Buffer, pH 6)

- LKB1 antibody clone Ley37D/G6 (Santa Cruz Biotechnology Dallas, TX) is a murine monoclonal IgG2b antibody anti-human LKB1, raised against recombinant LKB1 of human origin targeting an epitope located at the C-terminal region of the protein
- pAMPKα antibody (Thr172; Cell SignalingTechnology, Danvers, MA) is a Rabbit monoclonal antibody which detects endogenous AMPKα1 only when phosphorylated at threonine 183 and endogenous AMPKα2 only when phosphorylated at threonine 172. The antibody does not detect the regulatory β or γ subunits.
