# Supplementary material for: LKB1 Loss Correlates with STING Loss and, in Cooperation with β-Catenin Membranous Loss, Indicates Poor Prognosis in Patients with Operable Non-Small Cell Lung Cancer
Source: Cancers (Basel). 2024 May 10;16(10):1818. doi: 10.3390/cancers16101818 (PMC11120022; doi:10.3390/cancers16101818)
Supplement: Supplementary file 1 [file cancers-16-01818-s001.zip › Supplementary material S2.pdf]

Supplementary material 2 (S2)

Assessment of immunohistochemical expression

LKB1 and pAMPK: Only cancer cells were scored. Evaluation was based on the range of cytoplasmic staining intensity using a four scoring scale (0-3). 0=no appreciable staining, 1=very low staining, 2=strong staining and 3=very strong staining. LKB1 expression was considered as "intact" if any appreciable level of staining intensity was present [H-score > 0] and as "lost" when there was no appreciable level of staining intensity [H-score = 0] [37]. Interestingly, both LKB1 and pAMPK showed a very broad range of staining intensities [41]. Ciliated bronchial epithelium served as internal positive control of LKB1, as LKB1was highly expressed in the apical surface (i.e. cilia), consistent with LKB1 known roles in the establishment and maintenance of epithelial polarity. Cases with intact LKB1 staining, showed different degrees of staining, varying from weak to strong cytoplasmic staining. Tumors with heterogeneous staining, composed of areas of intact and lost staining, were classified as LKB1 intact [36,41,42].

PD-L1: Was evaluated in tumor cells. PD-L1 expression was considered as "negative" if positively stained tumor cells were ≤ 1% , as "low positive" if positive tumor cells were ≥ 1% and 49% and as "high positive" if the percentage of positively stained were ≥ 50%. [PD-L1 negative (PD-L1 < 1%), low positive (1% ≤ PD-L1 < 50%), or high positive (PD-L1 ≥ 50%)] [37]

STING: Was analyzed by estimating the percentage of tumor cells at each staining intensity level (0=negative, 1=low, 2=intermediate and 3= high) and subsequently a Histo-score was assigned using the formula: [0 X (% tumor epithelium 0+) +1 X (% of tumor cells 1+) +2 X (% of tumor cells 2+) + 3 X (% of tumor cells 3+)]. A score ranging from 0-300 was assigned and then using the median value expression was appointed as high (> median value) and low (≤ median value) expression [43].

ZEB-1: Normal productive phase endometrium was used as positive control, and only nuclear staining was considered positive. Staining was scored semiquantitavely using the Histoscore (H-SCORE) scoring system derived by summing the percentages of cells staining at each intensity (0-3) multiplied

by the weighted intensity of staining. The median was used as a cut off for high (> median value) and low (≤ median value) expression [34].

p53: p53 was assessed in tumor cells. Nuclear staining only was evaluated as positive, since it it has long been recognized that nonsynonymous *TP53* missense mutations result in nuclear accumulation of p53 protein that can be detected as overexpression by immunohistochemistry. As "mutation-type p53 immunohistochemical expression patterns", correlating with the presence of a *TP53* mutation, were considered 2 patterns. One was the "complete absence" defined as 100% absent nuclear expression (always confronting with internal positive control cells as lymphocytes, fibroblasts, endothelial cells and the other, the "overexpression" pattern defined as diffuse strong nuclear positivity involving ≥ 80% of the tumor cells -but usually almost 100%. In the presence of immunohistochemical staining with an admixture of negative cells, weakly and strongly positive cells, the tumor was characterized as wild-type. Wild-type staining is characterized by an admixture of negative cells, weakly and strongly positive cells [44].

p16: Only nuclear staining was evaluated. p16, was considered as downregulated ("low") if the percentage of the positively stained cells is <10 %. Adjacent stroma fibroblasts served as internal positive control [47] .

Cyclin D1 : Only nuclear staining was assessed. Cyclin D1, was considered as upregulated ("high") if the percentage of the positively stained tumor cells was >5 %. Adjacent stroma fibroblasts served as internal positive control [47].

PDGFRα, PDGFRβ: Tissue sections were scored for cytoplasmic staining in a semiquantitative fashion. For tumor cells expression, a value designated as the H-SCORE was derived by summing the percentages of cells staining at each intensity (0-3) multiplied by the weighted intensity of staining. The median was used as a cut off for high and low expression [48].

CD24: The strong membranous staining of the pneumonocytes of normal lung parenchyma was used as internal positive control. Staining was semiquantitatively scored (0, 1+, 2+, 3+) and grouped into high (2+, 3+) and low (0, 1+) level expression [49].

VEGFC: Scores were calculated by multiplying the staining intensity and extension at each intensity level. The extension of the cytoplasmic staining observed was scored as the percentage of positive cells (0% to 100%), and the intensity of staining was assessed by comparison with a known external positive control (0: below the level of detection; 1: weak; 2: moderate; and 3: strong). Median values were used as the cutoff [50].

β-catenin: The percentage of the tumor cells stained with complete membrane staining was evaluated and scored as "lost" (0): in the presence of ≤5% of tumor cells with preserved membrane staining, as "low" (1): in the presence 6-25% tumors cells with preserved membrane staining, as "moderate" (2): when 26≤70 % of the tumor cells preserve the membrane staining, and as "high" (3) when >70% of the tumor cells. Patchy, heterogeneous areas of various grades of retained membrane staining was observed in the majority of the tumors [51].
