# Supplementary material for: LKB1 Loss Correlates with STING Loss and, in Cooperation with β-Catenin Membranous Loss, Indicates Poor Prognosis in Patients with Operable Non-Small Cell Lung Cancer
Source: Cancers (Basel). 2024 May 10;16(10):1818. doi: 10.3390/cancers16101818 (PMC11120022; doi:10.3390/cancers16101818)
Supplement: Supplementary file 1 [file cancers-16-01818-s001.zip › Supplementary material S3.pdf]

Sequencies of the primers and Taqman probes

LKB1
FORWARD PRIMER
5'- CGGCCAAGAGGTTCTCCAT-3'
REVERSE PRIMER
5'- GCTTCAGCCGGAGGATGTT-3'
TAQMAN PROBE
5'-FAM- CACAGCTGGTTCCGGA-BBQ-3'

NEDD9

FORWARD PRIMER
5′-GGGTAAAAAGGTGATAACCCCCGT-3
REVERSE PRIMER
5′TGCTGATGAGGGAGGGATGTCGT-3
TAQMAN PROBE
5'-FAM- CAAGGGCCTTAT ATGAC -BBQ-3'

PGK1 FORWARD PRIMER 5'- GGCTGGATGGGCTTGGA-3'
PGK1 REVERSE PRIMER 5'-TCTGCTTAGCCCGAGTGACA-3'
PGK1 Probe 5'-FAM-TGTGGTCCTGAAAGCAGCAAGAAGTATGC-3'MGB

β-actin
FORWARD PRIMER 5′-GGC ACC CAG CAC AAT GAA G-3′
Probe 5'-FAM-TCA AGA TCA TTG CTC CTC CTG AGC GC--3 '
REVERSE PRIMER 5′-GCC GAT CCA CAC GGA GTA CT-3′
