# Supplementary material for: LKB1 Loss Correlates with STING Loss and, in Cooperation with β-Catenin Membranous Loss, Indicates Poor Prognosis in Patients with Operable Non-Small Cell Lung Cancer
Source: Cancers (Basel). 2024 May 10;16(10):1818. doi: 10.3390/cancers16101818 (PMC11120022; doi:10.3390/cancers16101818)
Supplement: Supplementary file 1 [file cancers-16-01818-s001.zip › Supplementary material S6.pdf]

OVERALL - KRAS & BRAF MUTATIONS

KRAS MUTATIONS (No 37)	
G12V	9
G13D	7
G12S	5
G12D	5
G12C	5
G12A	2
G23D	6
G13R	1
G12D+G23D	1

LN(+)= 35
LN(0)= 2

BRAF MUTATIONS (No 20)	
V600E	17
V600K	1
V600E+K601E	2

LN(+)= 16
LN(0)= 4
