# Supplementary material for: LKB1 Loss Correlates with STING Loss and, in Cooperation with β-Catenin Membranous Loss, Indicates Poor Prognosis in Patients with Operable Non-Small Cell Lung Cancer
Source: Cancers (Basel). 2024 May 10;16(10):1818. doi: 10.3390/cancers16101818 (PMC11120022; doi:10.3390/cancers16101818)
Supplement: Supplementary file 1 [file cancers-16-01818-s001.zip › Supplementary Table S10.pdf]

Table S10 LUACs - LKB1 loss vs LKB1 intact Laboratory Variables

**Variable**	**N**	**Overall**, N = 248[1]	**LKB1 LOSS**, N = 51[1]	**LKB1 INTACT**, N = 197[1]	**p-value**[2]	**q-value**[3]
**pAMPK_TUMOR**	248				**<0.001**	<0.001
0		51 (21%)	51 (100%)	0 (0%)		
1		197 (79%)	**0 (0%)**	197 (100%)		
**KL**	248				<0.001	<0.001
NO KL		232 (94%)	35 (69%)	197 (100%)		
KL		16 (6.5%)	16 (31%)	0 (0%)		
**L**	248				<0.001	<0.001
NO L		242 (98%)	45 (88%)	197 (100%)		
L		6 (2.4%)	6 (12%)	0 (0%)		
**KC**	248				**<0.001**	<0.001
NO KC		227 (92%)	39 (76%)	188 (95%)		
KC		21 (8.5%)	**12 (24%)**	9 (4.6%)		
**KRAS**	248				**<0.001**	0.001
0		211 (85%)	35 (69%)	176 (89%)		
1		37 (15%)	**16 (31%)**	21 (11%)		
**STING_TUMOR**	248				**<0.001**	0.003
0		123 (50%)	**36 (71%)**	87 (44%)		

**Variable**	**N**	**Overall**, N = 248[1]	**LKB1 LOSS**, N = 51[1]	**LKB1 INTACT**, N = 197[1]	**p-value**[2]	**q-value**[3]
1		125 (50%)	15 (29%)	110 (56%)		
**PDGFRb_TUMOR**	248				0.003	0.009
0		124 (50%)	35 (69%)	89 (45%)		
1		124 (50%)	16 (31%)	108 (55%)		
**LKB1_RNA_TUMOR**	248				0.003	0.009
0		124 (50%)	35 (69%)	89 (45%)		
1		124 (50%)	16 (31%)	108 (55%)		
**CD24**	248				0.009	0.026
0		113 (46%)	15 (29%)	98 (50%)		
1		135 (54%)	**36 (71%)**	99 (50%)		
**p53_TUMOR**	248				0.013	0.032
0		147 (59%)	38 (75%)	109 (55%)		
1		101 (41%)	13 (25%)	88 (45%)		
**ZEB1_TUMOR**	248				0.014	0.033
0		90 (36%)	26 (51%)	64 (32%)		
1		158 (64%)	25 (49%)	133 (68%)		
**PDGFRa_TUMOR**	248				0.024	0.051
0		116 (47%)	31 (61%)	85 (43%)		
1		132 (53%)	20 (39%)	112 (57%)		

**Variable**	**N**	**Overall**, N = 248[1]	**LKB1 LOSS**, N = 51[1]	**LKB1 INTACT**, N = 197[1]	**p-value**[2]	**q-value**[3]
**VEGFC**	248				0.042	0.081
0		134 (54%)	34 (67%)	100 (51%)		
1		114 (46%)	17 (33%)	97 (49%)		
**Cyclin-D1**	248				0.054	0.10
0		66 (27%)	19 (37%)	47 (24%)		
1		182 (73%)	32 (63%)	150 (76%)		
**BRAF_TUMOR**	244				0.15	0.2
0		224 (92%)	44 (86%)	180 (93%)		
1		20 (8.2%)	7 (14%)	13 (6.7%)		
**PD-L1_TUMOR_SCORE**	248				0.2	0.2
0		159 (64%)	37 (73%)	122 (62%)		
1		89 (36%)	14 (27%)	75 (38%)		
**ZEB1_TUMOR_STROMA**	248				0.2	0.3
0		121 (49%)	29 (57%)	92 (47%)		
1		127 (51%)	22 (43%)	105 (53%)		
**KPL**	248				0.2	0.3
NO KPL		247 (100%)	50 (98%)	197 (100%)		
KPL		1 (0.4%)	1 (2.0%)	0 (0%)		
**KP**	248				0.3	0.4

**Variable**	**N**	**Overall**, N = 248[1]	**LKB1 LOSS**, N = 51[1]	**LKB1 INTACT**, N = 197[1]	**p-value**[2]	**q-value**[3]
NO KP		234 (94%)	50 (98%)	184 (93%)		
KP		14 (5.6%)	1 (2.0%)	13 (6.6%)		
**PDGFRa_TUMOR_STROMA**	248				0.3	0.4
0		65 (26%)	16 (31%)	49 (25%)		
1		183 (74%)	35 (69%)	148 (75%)		
**p16**	248				0.6	0.7
0		71 (29%)	13 (25%)	58 (29%)		
1		177 (71%)	38 (75%)	139 (71%)		
**K**	248				0.6	0.7
NO K		244 (98%)	51 (100%)	193 (98%)		
K		4 (1.6%)	0 (0%)	4 (2.0%)		
**b-Catenin_TUMOR_MEMBRANOUS_SCORE**	248				>0.9	>0.9
2-3		128 (52%)	26 (51%)	102 (52%)		
0-1		120 (48%)	**25 (49%)**	**95 (48%)**		
**NEDD9_TUMOR**	248				>0.9	>0.9
0		123 (50%)	25 (49%)	98 (50%)		
1		125 (50%)	26 (51%)	99 (50%)		
**PDGFRb_TUMOR_STROMA**	248				>0.9	>0.9

**Variable**	**N**	**Overall**, N = 248[1]	**LKB1 LOSS**, N = 51[1]	**LKB1 INTACT**, N = 197[1]	**p-value**[2]	**q-value**[3]
0		43 (17%)	9 (18%)	34 (17%)		
1		205 (83%)	42 (82%)	163 (83%)		

[1]n (%)

[2]Pearson's Chi-squared test; Fisher's exact test

[3]False discovery rate correction for multiple testing
