# Supplementary material for: LKB1 Loss Correlates with STING Loss and, in Cooperation with β-Catenin Membranous Loss, Indicates Poor Prognosis in Patients with Operable Non-Small Cell Lung Cancer
Source: Cancers (Basel). 2024 May 10;16(10):1818. doi: 10.3390/cancers16101818 (PMC11120022; doi:10.3390/cancers16101818)
Supplement: Supplementary file 1 [file cancers-16-01818-s001.zip › Supplementary Table S11.pdf]

Table S11 LUACs & LKB1 loss vs LUACs & LKB1 intact _Laboratory Variables

**Variable**	**N**	**Overall LUACs**, N = 120[1]	**LUACs & LKB1 LOSS**, N = 41[1]	**LUACs & LKB1 INTACT**, N = 79[1]	**p-value**[2]	**q-value**[3]
**pAMPK_TUMOR**	120				<0.001	<0.001
0		41 (34%)	41 (100%)	0 (0%)		
1		79 (66%)	0 (0%)	79 (100%)		
**KL**	120				<0.001	<0.001
NO KL		105 (88%)	26 (63%)	79 (100%)		
KL		15 (13%)	15 (37%)	0 (0%)		
**STING_TUMOR**	120				<0.001	0.005
0		53 (44%)	27 (66%)	26 (33%)		
1		67 (56%)	14 (34%)	53 (67%)		
**PDGFRb_TUMOR**	120				0.004	0.020
0		60 (50%)	28 (68%)	32 (41%)		
1		60 (50%)	13 (32%)	47 (59%)		
**KC**	120				0.004	0.020
NO KC		103 (86%)	30 (73%)	73 (92%)		

**Variable**	**N**	**Overall LUACs**, N = 120[1]	**LUACs & LKB1 LOSS**, N = 41[1]	**LUACs & LKB1 INTACT**, N = 79[1]	**p-value**[2]	**q-value**[3]
KC		17 (14%)	11 (27%)	6 (7.6%)		
**KRAS**	120				0.008	0.031
0		93 (78%)	26 (63%)	67 (85%)		
1		27 (23%)	15 (37%)	12 (15%)		
**p53**	120				0.015	0.051
0		79 (66%)	33 (80%)	46 (58%)		
1		41 (34%)	8 (20%)	33 (42%)		
**VEGFC**	120				0.018	0.051
0		61 (51%)	27 (66%)	34 (43%)		
1		59 (49%)	14 (34%)	45 (57%)		
**b-Catenin_TUMOR_MEMBRANOUS**	120				**0.019**	**0.051**
2-3		73 (61%)	19 (46%)	54 (68%)		
0-1		47 (39%)	**22 (54%)**	**25 (32%)**		
**Cyclin-D1**	120				0.035	0.083
0		30 (25%)	15 (37%)	15 (19%)		

Variable	N	Overall LUACs, N = 120[1]	LUACs & LKB1 LOSS, N = 41[1]	LUACs & LKB1 INTACT, N = 79[1]	p-value[2]	q-value[3]
1		90 (75%)	26 (63%)	64 (81%)		
**LKB1_RNA_TUMOR**	120				0.047	0.10
0		70 (58%)	29 (71%)	41 (52%)		
1		50 (42%)	12 (29%)	38 (48%)		
**p16**	120				0.13	0.3
0		40 (33%)	10 (24%)	30 (38%)		
1		80 (67%)	31 (76%)	49 (62%)		
**ZEB1_TUMOR_STROMA**	120				0.2	0.3
0		72 (60%)	21 (51%)	51 (65%)		
1		48 (40%)	20 (49%)	28 (35%)		
**BRAF_TUMOR**	119				0.2	0.3
0		108 (91%)	35 (85%)	73 (94%)		
1		11 (9.2%)	6 (15%)	5 (6.4%)		
**PD-L1_TUMOR_SCORE**	120				0.3	0.4
0		83 (69%)	31 (76%)	52 (66%)		

Variable	N	Overall LUACs, N = 120[1]	LUACs & LKB1 LOSS, N = 41[1]	LUACs & LKB1 INTACT, N = 79[1]	p-value[2]	q-value[3]
1		37 (31%)	10 (24%)	27 (34%)		
**PDGFRa_TUMOR**	120				0.3	0.4
0		59 (49%)	23 (56%)	36 (46%)		
1		61 (51%)	18 (44%)	43 (54%)		
**KPL**	120				0.3	0.5
NO KPL		119 (99%)	40 (98%)	79 (100%)		
KPL		1 (0.8%)	1 (2.4%)	0 (0%)		
**ZEB1_TUMOR**	120				0.4	0.5
0		58 (48%)	22 (54%)	36 (46%)		
1		62 (52%)	19 (46%)	43 (54%)		
**KP**	120				0.4	0.5
NO KP		113 (94%)	40 (98%)	73 (92%)		
KP		7 (5.8%)	1 (2.4%)	6 (7.6%)		
**CD24**	120				0.5	0.6
0		31 (26%)	12 (29%)	19 (24%)		

Variable	N	Overall LUACs, N = 120[1]	LUACs & LKB1 LOSS, N = 41[1]	LUACs & LKB1 INTACT, N = 79[1]	p-value[2]	q-value[3]
1		89 (74%)	29 (71%)	60 (76%)		
**K**	120				0.5	0.6
NO K		118 (98%)	41 (100%)	77 (97%)		
K		2 (1.7%)	0 (0%)	2 (2.5%)		
**PDGFRb_TUMOR_STROMA**	120				0.7	0.7
0		26 (22%)	8 (20%)	18 (23%)		
1		94 (78%)	33 (80%)	61 (77%)		
**PDGFR_a_TUMOR_STROMA**	120				0.8	0.8
0		46 (38%)	15 (37%)	31 (39%)		
1		74 (62%)	26 (63%)	48 (61%)		
**NEDD9_TUMOR**	120				>0.9	>0.9
0		59 (49%)	20 (49%)	39 (49%)		
1		61 (51%)	21 (51%)	40 (51%)		

[1]n (%)

[2]Pearson's Chi-squared test; Fisher's exact test

[3]False discovery rate correction for multiple testing
