# Supplementary material for: LKB1 Loss Correlates with STING Loss and, in Cooperation with β-Catenin Membranous Loss, Indicates Poor Prognosis in Patients with Operable Non-Small Cell Lung Cancer
Source: Cancers (Basel). 2024 May 10;16(10):1818. doi: 10.3390/cancers16101818 (PMC11120022; doi:10.3390/cancers16101818)
Supplement: Supplementary file 1 [file cancers-16-01818-s001.zip › Supplementary Table S12.pdf]

Table S12 LUACs & LKB1 loss vs LUACs & LKB1 intact _Clinical Variables

**Variable**	**N**	**LUACs Overall**, N = 120[1]	**LUACs & LKB1 LOSS**, N = 41[1]	**LUACs & LKB1 INTACT**, N = 79[1]	**p-value**[2]	**q-value**[3]
**Tumor Size**	120				0.060	0.6
<=3cm		46 (38%)	11 (27%)	35 (44%)		
>3cm & <=5cm		38 (32%)	12 (29%)	26 (33%)		
5cm & <=7cm		17 (14%)	10 (24%)	7 (8.9%)		
>7cm		19 (16%)	8 (20%)	11 (14%)		
**ADCs_Micropapillary Component**	120				0.2	0.6
0%		95 (79%)	35 (85%)	60 (76%)		
<=5%		7 (5.8%)	2 (4.9%)	5 (6.3%)		
>5%		1 (0.8%)	1 (2.4%)	0 (0%)		
>=10%		17 (14%)	3 (7.3%)	14 (18%)		
**META STATUS**	120				0.3	0.6
LN META-		31 (26%)	8 (20%)	23 (29%)		
LN META+		89 (74%)	33 (80%)	56 (71%)		

Variable	N	LUACs Overall, N = 120[1]	LUACs & LKB1 LOSS, N = 41[1]	LUACs & LKB1 INTACT, N = 79[1]	p-value[2]	q-value[3]
**pSTAGE**	120				0.3	0.6
IA		7 (5.8%)	2 (4.9%)	5 (6.3%)		
IB		12 (10%)	2 (4.9%)	10 (13%)		
IIA		19 (16%)	5 (12%)	14 (18%)		
IIB		19 (16%)	10 (24%)	9 (11%)		
IIIA		61 (51%)	21 (51%)	40 (51%)		
IIIB		1 (0.8%)	0 (0%)	1 (1.3%)		
IV		1 (0.8%)	1 (2.4%)	0 (0%)		
**ADCs_Secondary Histological Pattern**	120				0.3	0.6
NO SECONDARY		42 (35%)	19 (46%)	23 (29%)		
LEPIDIC		6 (5.0%)	2 (4.9%)	4 (5.1%)		
ACINAR		31 (26%)	10 (24%)	21 (27%)		
PAPILLARY		17 (14%)	3 (7.3%)	14 (18%)		
MICROPAPILLARY		15 (13%)	3 (7.3%)	12 (15%)		
SOLID		9 (7.5%)	4 (9.8%)	5 (6.3%)		

Variable	N	LUACs Overall, N = 120[1]	LUACs & LKB1 LOSS, N = 41[1]	LUACs & LKB1 INTACT, N = 79[1]	p-value[2]	q-value[3]
**HISTOTYPE**	120				0.3	0.6
ADC		110 (92%)	36 (88%)	74 (94%)		
Pleo ADC		10 (8.3%)	5 (12%)	5 (6.3%)		
**ADCs_Predominant Histological Pattern**	120				0.5	0.8
LEPIDIC		5 (4.2%)	1 (2.4%)	4 (5.1%)		
ACINAR		30 (25%)	8 (20%)	22 (28%)		
PAPILLARY		12 (10%)	4 (9.8%)	8 (10%)		
MICROPAPILLARY		1 (0.8%)	0 (0%)	1 (1.3%)		
SOLID		62 (52%)	27 (66%)	35 (44%)		
INVASIVE MUCINOUS		3 (2.5%)	0 (0%)	3 (3.8%)		
COLLOID		3 (2.5%)	0 (0%)	3 (3.8%)		
ENTERIC		4 (3.3%)	1 (2.4%)	3 (3.8%)		
**LN_STATUS**	120				0.6	0.8
LN0		31 (26%)	8 (20%)	23 (29%)		
LN1		42 (35%)	17 (41%)	25 (32%)		

**Variable**	**N**	**LUACs Overall**, N = 120[1]	**LUACs & LKB1 LOSS**, N = 41[1]	**LUACs & LKB1 INTACT**, N = 79[1]	**p-value**[2]	**q-value**[3]
LN2		13 (11%)	3 (7.3%)	10 (13%)		
LN3		2 (1.7%)	1 (2.4%)	1 (1.3%)		
LN1 & LN2		32 (27%)	12 (29%)	20 (25%)		
**GENDER**	120				0.6	0.8
MALE		97 (81%)	32 (78%)	65 (82%)		
FEMALE		23 (19%)	9 (22%)	14 (18%)		
**AGE_AT_DIAGNOSIS**	120				0.7	0.8
<70		97 (81%)	34 (83%)	63 (80%)		
>=70		23 (19%)	7 (17%)	16 (20%)		
**GRADE**	120				0.7	0.8
G1		12 (10%)	4 (9.8%)	8 (10%)		
G2		35 (29%)	10 (24%)	25 (32%)		
G3		73 (61%)	27 (66%)	46 (58%)		
**pSTAGE_binary**	120				0.9	0.9
IIIa - IV		63 (53%)	22 (54%)	41 (52%)		

**Variable**	**N**	**LUACs Overall**, N = 120[1]	**LUACs & LKB1 LOSS**, N = 41[1]	**LUACs & LKB1 INTACT**, N = 79[1]	**p-value**[2]	**q-value**[3]
I & II		57 (48%)	19 (46%)	38 (48%)		

[1]n (%)

[2]Pearson's Chi-squared test; Fisher's exact test

[3]False discovery rate correction for multiple testing
