# Supplementary material for: LKB1 Loss Correlates with STING Loss and, in Cooperation with β-Catenin Membranous Loss, Indicates Poor Prognosis in Patients with Operable Non-Small Cell Lung Cancer
Source: Cancers (Basel). 2024 May 10;16(10):1818. doi: 10.3390/cancers16101818 (PMC11120022; doi:10.3390/cancers16101818)
Supplement: Supplementary file 1 [file cancers-16-01818-s001.zip › Supplementary Table S13.pdf]

Table S13 Overall LN+ - Metastatic tumors with LKB1 loss vs Metastatic tumors with LKB1 intact - Laboratory Variables

**Variable**	**N**	**LN+ Overall**, N = 188[1]	**LKB1 LOSS & LN META+**, N = 41[1]	**LKB1 INTACT & LN META+**, N = 147[1]	**p-value**[2]	**q-value**[3]
**pAMPK_TUMOR**	188				<0.001	<0.001
0		41 (22%)	41 (100%)	0 (0%)		
1		147 (78%)	0 (0%)	147 (100%)		
**KL**	188				<0.001	<0.001
NO KL		172 (91%)	25 (61%)	147 (100%)		
KL		16 (8.5%)	16 (39%)	0 (0%)		
**PDGFRb_TUMOR**	188				<0.001	<0.001
0		72 (38%)	27 (66%)	45 (31%)		
1		116 (62%)	14 (34%)	102 (69%)		
**KC**	188				<0.001	<0.001
NO KC		168 (89%)	29 (71%)	139 (95%)		
KC		20 (11%)	12 (29%)	8 (5.4%)		
**KRAS**	188				<0.001	<0.001
0		153 (81%)	25 (61%)	128 (87%)		
1		35 (19%)	16 (39%)	19 (13%)		
**ZEB1_TUMOR**	188				0.003	0.012
0		45 (24%)	17 (41%)	28 (19%)		
1		143 (76%)	24 (59%)	119 (81%)		

**Variable**	**N**	**LN+ Overall**, N = 188[1]	**LKB1 LOSS & LN META+**, N = 41[1]	**LKB1 INTACT & LN META+**, N = 147[1]	**p-value**[2]	**q-value**[3]
**LKB1_RNA_TUMOR**	188				0.008	0.029
0		94 (50%)	28 (68%)	66 (45%)		
1		94 (50%)	13 (32%)	81 (55%)		
**L**	188				0.010	0.031
NO L		185 (98%)	38 (93%)	147 (100%)		
L		3 (1.6%)	3 (7.3%)	0 (0%)		
**STING_TUMOR**	188				**0.014**	0.035
0		101 (54%)	**29 (71%)**	**72 (49%)**		
1		87 (46%)	12 (29%)	75 (51%)		
**PDGFRa_TUMOR**	188				0.014	0.035
0		83 (44%)	25 (61%)	58 (39%)		
1		105 (56%)	16 (39%)	89 (61%)		
**CD24**	188				0.025	0.056
0		84 (45%)	12 (29%)	72 (49%)		
1		104 (55%)	29 (71%)	75 (51%)		
**p53**	188				0.031	0.065
0		110 (59%)	30 (73%)	80 (54%)		
1		78 (41%)	11 (27%)	67 (46%)		
**VEGFC**	188				0.036	0.069
0		83 (44%)	24 (59%)	59 (40%)		
1		105 (56%)	17 (41%)	88 (60%)		

Variable	N	LN+ Overall, N = 188[1]	LKB1 LOSS & LN META+, N = 41[1]	LKB1 INTACT & LN META+, N = 147[1]	p-value[2]	q-value[3]
**Cyclin-D1**	188				0.064	0.12
0		56 (30%)	17 (41%)	39 (27%)		
1		132 (70%)	24 (59%)	108 (73%)		
**ZEB1_TUMOR_STROMA**	188				0.2	0.3
0		71 (38%)	19 (46%)	52 (35%)		
1		117 (62%)	22 (54%)	95 (65%)		
**KPL**	188				0.2	0.3
NO KPL		187 (99%)	40 (98%)	147 (100%)		
KPL		1 (0.5%)	1 (2.4%)	0 (0%)		
**PD-L1_TUMOR_SCORE**	188				0.3	0.4
0		124 (66%)	30 (73%)	94 (64%)		
1		64 (34%)	11 (27%)	53 (36%)		
**KP**	188				0.3	0.4
NO KP		175 (93%)	40 (98%)	135 (92%)		
KP		13 (6.9%)	1 (2.4%)	12 (8.2%)		
**p16**	188				0.4	0.4
0		52 (28%)	9 (22%)	43 (29%)		
1		136 (72%)	32 (78%)	104 (71%)		
**BRAF_TUMOR**	184				0.4	0.4
0		168 (91%)	36 (88%)	132 (92%)		
1		16 (8.7%)	5 (12%)	11 (7.7%)		

**Variable**	**N**	**LN+ Overall**, N = 188[1]	**LKB1 LOSS & LN META+**, N = 41[1]	**LKB1 INTACT & LN META+**, N = 147[1]	**p-value**[2]	**q-value**[3]
**PDGFRa_TUMOR_STROMA**	188				0.5	0.6
0		47 (25%)	12 (29%)	35 (24%)		
1		141 (75%)	29 (71%)	112 (76%)		
**PDGFRb_TUMOR_STROMA**	188				0.6	0.7
0		22 (12%)	6 (15%)	16 (11%)		
1		166 (88%)	35 (85%)	131 (89%)		
**b-Catenin_TUMOR_MEMBRANOUS**	188				0.8	0.8
2-3		82 (44%)	17 (41%)	65 (44%)		
0-1		106 (56%)	24 (59%)	82 (56%)		
**NEDD9_TUMOR**	188				>0.9	>0.9
0		96 (51%)	21 (51%)	75 (51%)		
1		92 (49%)	20 (49%)	72 (49%)		
**K**	188				>0.9	>0.9
NO K		185 (98%)	41 (100%)	144 (98%)		
K		3 (1.6%)	0 (0%)	3 (2.0%)		

[1]n (%)

[2]Pearson's Chi-squared test; Fisher's exact test

[3]False discovery rate correction for multiple testing
