# Supplementary material for: LKB1 Loss Correlates with STING Loss and, in Cooperation with β-Catenin Membranous Loss, Indicates Poor Prognosis in Patients with Operable Non-Small Cell Lung Cancer
Source: Cancers (Basel). 2024 May 10;16(10):1818. doi: 10.3390/cancers16101818 (PMC11120022; doi:10.3390/cancers16101818)
Supplement: Supplementary file 1 [file cancers-16-01818-s001.zip › Supplementary Table S14.pdf]

Table S14 LUACs LN+ - Metastatic tumors with LKB1 loss vs Metastatic tumors with LKB1 intact - Laboratory Variables

Variable	N	LUACs LN(+) Overall, N = 89[1]	LUACs & LKB1 LOSS & LN META+, N = 33[1]	LUACs & LKB1 INTACT & LN META+, N = 56[1]	p-value[2]	q-value[3]
**pAMPK_TUMOR**	89				<0.001	<0.001
0		33 (37%)	33 (100%)	0 (0%)		
1		56 (63%)	**0 (0%)**	**56 (100%)**		
**KL**	89				<0.001	<0.001
NO KL		74 (83%)	18 (55%)	56 (100%)		
KL		15 (17%)	15 (45%)	0 (0%)		
**PDGFRb_TUMOR**	89				<0.001	<0.001
0		35 (39%)	22 (67%)	13 (23%)		
1		54 (61%)	11 (33%)	43 (77%)		
**STING_TUMOR**	89				**0.005**	0.030
0		42 (47%)	**22 (67%)**	**20 (36%)**		
1		47 (53%)	11 (33%)	36 (64%)		
**b-Catenin_TUMOR_MEMBRANOUS**	89				**0.007**	0.033
2-3		49 (55%)	12 (36%)	37 (66%)		
0-1		40 (45%)	**21 (64%)**	**19 (34%)**		
**KC**	89				**0.009**	0.035
NO KC		72 (81%)	22 (67%)	50 (89%)		
KC		17 (19%)	**11 (33%)**	**6 (11%)**		

**Variable**	**N**	**LUACs LN(+) Overall**, N = 89[1]	**LUACs & LKB1 LOSS & LN META+**, N = 33[1]	**LUACs & LKB1 INTACT & LN META+**, N = 56[1]	**p-value**[2]	**q-value**[3]
**KRAS**	89				0.010	0.035
0		63 (71%)	18 (55%)	45 (80%)		
1		26 (29%)	15 (45%)	11 (20%)		
**p53**	89				0.017	0.052
0		59 (66%)	27 (82%)	32 (57%)		
1		30 (34%)	6 (18%)	24 (43%)		
**VEGFC**	89				0.019	0.052
0		37 (42%)	19 (58%)	18 (32%)		
1		52 (58%)	14 (42%)	38 (68%)		
**Cyclin-D1**	89				0.025	0.062
0		23 (26%)	13 (39%)	10 (18%)		
1		66 (74%)	20 (61%)	46 (82%)		
**L**	89				0.048	0.11
NO L		86 (97%)	30 (91%)	56 (100%)		
L		3 (3.4%)	3 (9.1%)	0 (0%)		
**LKB1_RNA_TUMOR**	89				0.10	0.2
0		52 (58%)	**23 (70%)**	**29 (52%)**		
1		37 (42%)	10 (30%)	27 (48%)		
**ZEB1_TUMOR**	89				0.11	0.2
0		31 (35%)	15 (45%)	16 (29%)		

Variable	N	**LUACs LN(+) Overall**, N = 89[1]	**LUACs & LKB1 LOSS & LN META+**, N = 33[1]	**LUACs & LKB1 INTACT & LN META+**, N = 56[1]	p-value[2]	q-value[3]
1		58 (65%)	18 (55%)	40 (71%)		
**PDGFRa_TUMOR**	89				0.13	0.2
0		42 (47%)	19 (58%)	23 (41%)		
1		47 (53%)	14 (42%)	33 (59%)		
**KP**	89				0.3	0.4
NO KP		82 (92%)	32 (97%)	50 (89%)		
KP		7 (7.9%)	1 (3.0%)	6 (11%)		
**p16**	89				0.3	0.4
0		28 (31%)	8 (24%)	20 (36%)		
1		61 (69%)	25 (76%)	36 (64%)		
**ZEB1_TUMOR_STROMA**	89				0.3	0.5
0		41 (46%)	13 (39%)	28 (50%)		
1		48 (54%)	20 (61%)	28 (50%)		
**CD24**	89				0.3	0.5
0		22 (25%)	10 (30%)	12 (21%)		
1		67 (75%)	23 (70%)	44 (79%)		
**KPL**	89				0.4	0.5
NO KPL		88 (99%)	32 (97%)	56 (100%)		
KPL		1 (1.1%)	1 (3.0%)	0 (0%)		
**NEDD9_RNA _TUMOR**	89				0.4	0.5

Variable	N	LUACs LN(+) Overall, N = 89[1]	LUACs & LKB1 LOSS & LN META+, N = 33[1]	LUACs & LKB1 INTACT & LN META+, N = 56[1]	p-value[2]	q-value[3]
0		41 (46%)	17 (52%)	24 (43%)		
1		48 (54%)	16 (48%)	32 (57%)		
**BRAF_TUMOR**	88				0.5	0.6
0		80 (91%)	29 (88%)	51 (93%)		
1		8 (9.1%)	4 (12%)	4 (7.3%)		
**PD-L1_TUMOR_SCORE_TPS**	89				0.5	0.6
0		64 (72%)	25 (76%)	39 (70%)		
1		25 (28%)	8 (24%)	17 (30%)		
**PDGFRa_TUMOR_STROMA**	89				0.8	0.9
0		34 (38%)	12 (36%)	22 (39%)		
1		55 (62%)	21 (64%)	34 (61%)		
**PDGFRb_TUMOR_STROMA**	89				0.9	>0.9
0		17 (19%)	6 (18%)	11 (20%)		
1		72 (81%)	27 (82%)	45 (80%)		
**K**	89				>0.9	>0.9
NO K		88 (99%)	33 (100%)	55 (98%)		
K		1 (1.1%)	0 (0%)	1 (1.8%)		

[1]n (%)

[2]Pearson's Chi-squared test; Fisher's exact test

Variable	N	**LUACs LN(+) Overall**, N = 89[1]	**LUACs & LKB1 LOSS & LN META+**, N = 33[1]	**LUACs & LKB1 INTACT & LN META+**, N = 56[1]	**p-value**[2]	**q-value**[3]

[3]False discovery rate correction for multiple testing
