# Supplementary material for: LKB1 Loss Correlates with STING Loss and, in Cooperation with β-Catenin Membranous Loss, Indicates Poor Prognosis in Patients with Operable Non-Small Cell Lung Cancer
Source: Cancers (Basel). 2024 May 10;16(10):1818. doi: 10.3390/cancers16101818 (PMC11120022; doi:10.3390/cancers16101818)
Supplement: Supplementary file 1 [file cancers-16-01818-s001.zip › Supplementary Table S15.pdf]

Table S15 LSCCs LN+ – Metastatic tumors with LKB1 loss vs Metastatic tumors with LKB1 intact – Laboratory Variables

Variable	N	LSCCs Overall, N = 95[1]	LSCC & LKB1 LOSS & LN META+, N = 6[1]	LSCC & LKB1 INTACT & LN META+, N = 89[1]	p-value[2]	q-value[3]
**pAMPK_TUMOR**	95				<0.001	<0.001
0		6 (6.3%)	6 (100%)	0 (0%)		
1		89 (94%)	0 (0%)	89 (100%)		
**ZEB1_TUMOR_STROMA**	95				0.053	0.7
0		27 (28%)	4 (67%)	23 (26%)		
1		68 (72%)	2 (33%)	66 (74%)		
**b-Catenin_TUMOR_MEMBRANOUS**	95				0.2	0.7
2-3		32 (34%)	4 (67%)	28 (31%)		
0-1		63 (66%)	2 (33%)	61 (69%)		
**CD24**	95				0.2	0.7
0		61 (64%)	2 (33%)	59 (66%)		
1		34 (36%)	4 (67%)	30 (34%)		
**PDGFRb_TUMOR**	95				0.2	0.7
0		35 (37%)	4 (67%)	31 (35%)		

**Variable**	**N**	**LSCCs Overall**, N = 95[1]	**LSCC & LKB1 LOSS & LN META+**, N = 6[1]	**LSCC & LKB1 INTACT & LN META+**, N = 89[1]	**p-value**[2]	**q-value**[3]
1		60 (63%)	2 (33%)	58 (65%)		
**PDGFRa_TUMOR**	95				0.2	0.7
0		37 (39%)	4 (67%)	33 (37%)		
1		58 (61%)	2 (33%)	56 (63%)		
**ZEB1_TUMOR**	95				0.2	0.7
0		14 (15%)	2 (33%)	12 (13%)		
1		81 (85%)	4 (67%)	77 (87%)		
**LKB1_RNA_TUMOR**	95				0.2	0.7
0		39 (41%)	4 (67%)	35 (39%)		
1		56 (59%)	2 (33%)	54 (61%)		
**STING_TUMOR**	95				0.4	>0.9
0		56 (59%)	5 (83%)	51 (57%)		
1		39 (41%)	1 (17%)	38 (43%)		
**p53**	95				0.4	>0.9
0		50 (53%)	2 (33%)	48 (54%)		

Variable	N	LSCCs Overall, N = 95[1]	LSCC & LKB1 LOSS & LN META+, N = 6[1]	LSCC & LKB1 INTACT & LN META+, N = 89[1]	p-value[2]	q-value[3]
1		45 (47%)	4 (67%)	41 (46%)		
**BRAF_TUMOR**	92				0.4	>0.9
0		84 (91%)	5 (83%)	79 (92%)		
1		8 (8.7%)	1 (17%)	7 (8.1%)		
**KP**	95				>0.9	>0.9
NO KP		90 (95%)	6 (100%)	84 (94%)		
KP		5 (5.3%)	0 (0%)	5 (5.6%)		
**p16**	95				>0.9	>0.9
0		23 (24%)	1 (17%)	22 (25%)		
1		72 (76%)	5 (83%)	67 (75%)		
**Cyclin-D1**	95				>0.9	>0.9
0		30 (32%)	2 (33%)	28 (31%)		
1		65 (68%)	4 (67%)	61 (69%)		
**VEGFC**	95				>0.9	>0.9
0		43 (45%)	3 (50%)	40 (45%)		

Variable	N	**LSCCs Overall**, N = 95[1]	**LSCC & LKB1 LOSS & LN META+**, N = 6[1]	**LSCC & LKB1 INTACT & LN META+**, N = 89[1]	p-value[2]	q-value[3]
1		52 (55%)	3 (50%)	49 (55%)		
**PD-L1_TUMOR_SCORE**	95				>0.9	>0.9
0		58 (61%)	4 (67%)	54 (61%)		
1		37 (39%)	2 (33%)	35 (39%)		
**KRAS**	95				>0.9	>0.9
0		88 (93%)	6 (100%)	82 (92%)		
1		7 (7.4%)	0 (0%)	7 (7.9%)		
**PDGFRa_TUMOR_STROMA**	95				>0.9	>0.9
0		12 (13%)	0 (0%)	12 (13%)		
1		83 (87%)	6 (100%)	77 (87%)		
**PDGFRb_TUMOR_STROMA**	95				>0.9	>0.9
0		4 (4.2%)	0 (0%)	4 (4.5%)		
1		91 (96%)	6 (100%)	85 (96%)		
**NEDD9_TUMOR**	95				>0.9	>0.9
0		53 (56%)	3 (50%)	50 (56%)		

**Variable**	**N**	**LSCCs Overall**, N = 95[1]	**LSCC & LKB1 LOSS & LN META+**, N = 6[1]	**LSCC & LKB1 INTACT & LN META+**, N = 89[1]	**p-value**[2]	**q-value**[3]
1		42 (44%)	3 (50%)	39 (44%)		
**K**	95				>0.9	>0.9
NO K		93 (98%)	6 (100%)	87 (98%)		
K		2 (2.1%)	0 (0%)	2 (2.2%)		
**KPL**	95				>0.9	>0.9
NO KPL		95 (100%)	6 (100%)	89 (100%)		
KPL		0 (0%)	0 (0%)	0 (0%)		
**KL**	95				>0.9	>0.9
NO KL		95 (100%)	6 (100%)	89 (100%)		
KL		0 (0%)	0 (0%)	0 (0%)		
**KC**	95				>0.9	>0.9
NO KC		93 (98%)	6 (100%)	87 (98%)		
KC		2 (2.1%)	0 (0%)	2 (2.2%)		
**L**	95				>0.9	>0.9
NO L		95 (100%)	6 (100%)	89 (100%)		

**Variable**	**N**	**LSCCs Overall**, N = 95[1]	**LSCC & LKB1 LOSS & LN META+**, N = 6[1]	**LSCC & LKB1 INTACT & LN META+**, N = 89[1]	**p-value**[2]	**q-value**[3]
L		0 (0%)	0 (0%)	0 (0%)		

[1]n (%)

[2]Fisher's exact test

[3]False discovery rate correction for multiple testing
