# Supplementary material for: LKB1 Loss Correlates with STING Loss and, in Cooperation with β-Catenin Membranous Loss, Indicates Poor Prognosis in Patients with Operable Non-Small Cell Lung Cancer
Source: Cancers (Basel). 2024 May 10;16(10):1818. doi: 10.3390/cancers16101818 (PMC11120022; doi:10.3390/cancers16101818)
Supplement: Supplementary file 1 [file cancers-16-01818-s001.zip › Supplementary Table S16.pdf]

Table S16 Co-mutational Cohorts - Laboratory Variables

Variable	N	Overall, N = 83[1]	KRAS MUTANT & LKB1 INTACT, N = 21[1]	K N = 4[1]	KC N = 21[1]	KL N = 16[1]	KP N = 14[1]	KPL N = 1[1]	L N = 6[1]	p-value[2]	q-value[3]
**pAMPK**	83									<0.001	<0.001
0		36 (43%)	0 (0%)	0 (0%)	12 (57%)	16 (100%)	1 (7.1%)	1 (100%)	6 (100%)		
1		47 (57%)	21 (100%)	4 (100%)	9 (43%)	0 (0%)	13 (93%)	0 (0%)	0 (0%)		
**VEGFC**	83									0.005	0.041
0		31 (37%)	5 (24%)	0 (0%)	9 (43%)	8 (50%)	3 (21%)	0 (0%)	6 (100%)		
1		52 (63%)	16 (76%)	4 (100%)	12 (57%)	8 (50%)	11 (79%)	1 (100%)	0 (0%)		
**STING**	83									0.023	0.12
0		36 (43%)	6 (29%)	2 (50%)	7 (33%)	9 (56%)	5 (36%)	1 (100%)	6 (100%)		
1		47 (57%)	15 (71%)	2 (50%)	14 (67%)	7 (44%)	9 (64%)	0 (0%)	0 (0%)		
**ZEB1 TUMOR**	83									0.031	0.12
0		23 (28%)	3 (14%)	2 (50%)	6 (29%)	7 (44%)	1 (7.1%)	0 (0%)	4 (67%)		
1		60 (72%)	18 (86%)	2 (50%)	15 (71%)	9 (56%)	13 (93%)	1 (100%)	2 (33%)		
**PDGFRa TUMOR**	83									0.13	0.4

**Variable**	**N**	**Overall**, N = 83[1]	**KRAS MUTANT & LKB1 INTACT**, N = 21[1]	**K** N = 4[1]	**KC** N = 21[1]	**KL** N = 16[1]	**KP** N = 14[1]	**KPL** N = 1[1]	**L** N = 6[1]	**p-value**[2]	**q-value**[3]
0		38 (46%)	6 (29%)	1 (25%)	9 (43%)	10 (63%)	6 (43%)	1 (100%)	5 (83%)		
1		45 (54%)	15 (71%)	3 (75%)	12 (57%)	6 (38%)	8 (57%)	0 (0%)	1 (17%)		
**ZEB1_TUMOR_STROMA**	83									0.14	0.4
0		24 (29%)	4 (19%)	1 (25%)	6 (29%)	6 (38%)	2 (14%)	1 (100%)	4 (67%)		
1		59 (71%)	17 (81%)	3 (75%)	15 (71%)	10 (63%)	12 (86%)	0 (0%)	2 (33%)		
**PDGFRb_TUMOR_STROMA**	83									0.2	0.4
0		9 (11%)	1 (4.8%)	0 (0%)	2 (9.5%)	2 (13%)	1 (7.1%)	0 (0%)	3 (50%)		
1		74 (89%)	20 (95%)	4 (100%)	19 (90%)	14 (88%)	13 (93%)	1 (100%)	3 (50%)		
**PDGFR_a_TUMOR_STROMA**	83									0.2	0.4
0		16 (19%)	3 (14%)	0 (0%)	3 (14%)	3 (19%)	3 (21%)	1 (100%)	3 (50%)		
1		67 (81%)	18 (86%)	4 (100%)	18 (86%)	13 (81%)	11 (79%)	0 (0%)	3 (50%)		
**LKB1_RNA_TUMOR**	83									0.3	0.5
0		54 (65%)	12 (57%)	1 (25%)	17 (81%)	11 (69%)	9 (64%)	1 (100%)	3 (50%)		
1		29 (35%)	9 (43%)	3 (75%)	4 (19%)	5 (31%)	5 (36%)	0 (0%)	3 (50%)		
**PDGFRb_TUMOR**	83									0.3	0.5

Variable	N	Overall, N = 83[1]	KRAS MUTANT & LKB1 INTACT, N = 21[1]	K N = 4[1]	KC N = 21[1]	KL N = 16[1]	KP N = 14[1]	KPL N = 1[1]	L N = 6[1]	p-value[2]	q-value[3]
0		47 (57%)	11 (52%)	4 (100%)	10 (48%)	10 (63%)	7 (50%)	0 (0%)	5 (83%)		
1		36 (43%)	10 (48%)	0 (0%)	11 (52%)	6 (38%)	7 (50%)	1 (100%)	1 (17%)		
**Cyclin-D1**	83									0.3	0.5
0		23 (28%)	5 (24%)	1 (25%)	3 (14%)	5 (31%)	5 (36%)	1 (100%)	3 (50%)		
1		60 (72%)	16 (76%)	3 (75%)	18 (86%)	11 (69%)	9 (64%)	0 (0%)	3 (50%)		
**b-Catenin_TUMOR_MEMBRANOUS**	83									0.7	0.9
2-3		42 (51%)	11 (52%)	2 (50%)	13 (62%)	6 (38%)	6 (43%)	0 (0%)	4 (67%)		
0-1		41 (49%)	10 (48%)	2 (50%)	8 (38%)	10 (63%)	8 (57%)	1 (100%)	2 (33%)		
**CD24**	83									0.7	0.9
0		29 (35%)	7 (33%)	1 (25%)	6 (29%)	5 (31%)	7 (50%)	1 (100%)	2 (33%)		
1		54 (65%)	14 (67%)	3 (75%)	15 (71%)	11 (69%)	7 (50%)	0 (0%)	4 (67%)		
**PD-L1_TUMOR**	83									0.8	0.9
0		58 (70%)	14 (67%)	2 (50%)	15 (71%)	13 (81%)	10 (71%)	1 (100%)	3 (50%)		
1		25 (30%)	7 (33%)	2 (50%)	6 (29%)	3 (19%)	4 (29%)	0 (0%)	3 (50%)		
**NEDD9_TUMOR**	83									>0.9	>0.9

**Variable**	**N**	**Overall**, N = 83[1]	**KRAS MUTANT & LKB1 INTACT**, N = 21[1]	**K** N = 4[1]	**KC** N = 21[1]	**KL** N = 16[1]	**KP** N = 14[1]	**KPL** N = 1[1]	**L** N = 6[1]	**p-value**[2]	**q-value**[3]
0		42 (51%)	10 (48%)	3 (75%)	9 (43%)	9 (56%)	7 (50%)	1 (100%)	3 (50%)		
1		41 (49%)	11 (52%)	1 (25%)	12 (57%)	7 (44%)	7 (50%)	0 (0%)	3 (50%)		
**BRAF_TUMOR**	81									>0.9	>0.9
0		75 (93%)	18 (90%)	4 (100%)	18 (90%)	15 (94%)	13 (93%)	1 (100%)	6 (100%)		
1		6 (7.4%)	2 (10%)	0 (0%)	2 (10%)	1 (6.3%)	1 (7.1%)	0 (0%)	0 (0%)		

[1]n (%)

[2]Fisher's exact test

[3]False discovery rate correction for multiple testing
