# Supplementary material for: LKB1 Loss Correlates with STING Loss and, in Cooperation with β-Catenin Membranous Loss, Indicates Poor Prognosis in Patients with Operable Non-Small Cell Lung Cancer
Source: Cancers (Basel). 2024 May 10;16(10):1818. doi: 10.3390/cancers16101818 (PMC11120022; doi:10.3390/cancers16101818)
Supplement: Supplementary file 1 [file cancers-16-01818-s001.zip › Supplementary Table S17.pdf]

Table S17 Co-mutational Cohorts - Clinical Variables

Variable	N	Overall, N = 83[1]	KRAS MUTANT & LKB1 INTACT N = 21[1]	K N = 4[1]	KC N = 21[1]	KL N = 16[1]	KP N = 14[1]	KPL N = 1[1]	L N = 6[1]	p-value[2]	q-value[3]
**META STATUS**	83									0.032	0.3
LN META-		8 (9.6%)	2 (9.5%)	1 (25%)	1 (4.8%)	0 (0%)	1 (7.1%)	0 (0%)	3 (50%)		
LN META+		75 (90%)	19 (90%)	3 (75%)	20 (95%)	16 (100%)	13 (93%)	1 (100%)	3 (50%)		
**AGE_AT_DIAGNOSIS**	83									0.3	>0.9
<70		66 (80%)	15 (71%)	2 (50%)	18 (86%)	15 (94%)	10 (71%)	1 (100%)	5 (83%)		
>=70		17 (20%)	6 (29%)	2 (50%)	3 (14%)	1 (6.3%)	4 (29%)	0 (0%)	1 (17%)		
**ADCs_Micropapillary Component**	58									0.6	>0.9
0%		46 (79%)	9 (75%)	2 (100%)	14 (82%)	13 (87%)	4 (57%)	1 (100%)	3 (75%)		
<=5%		5 (8.6%)	1 (8.3%)	0 (0%)	2 (12%)	1 (6.7%)	1 (14%)	0 (0%)	0 (0%)		
>5%		2 (3.4%)	0 (0%)	0 (0%)	1 (5.9%)	1 (6.7%)	0 (0%)	0 (0%)	0 (0%)		
>=10%		5 (8.6%)	2 (17%)	0 (0%)	0 (0%)	0 (0%)	2 (29%)	0 (0%)	1 (25%)		
**GRADE**	83									0.7	>0.9
G1		4 (4.8%)	0 (0%)	0 (0%)	2 (9.5%)	2 (13%)	0 (0%)	0 (0%)	0 (0%)		
G2		25 (30%)	7 (33%)	1 (25%)	7 (33%)	5 (31%)	5 (36%)	0 (0%)	0 (0%)		
G3		54 (65%)	14 (67%)	3 (75%)	12 (57%)	9 (56%)	9 (64%)	1 (100%)	6 (100%)		

Variable	N	Overall, N = 83[1]	KRAS MUTANT & LKB1 INTACT N = 21[1]	K N = 4[1]	KC N = 21[1]	KL N = 16[1]	KP N = 14[1]	KPL N = 1[1]	L N = 6[1]	p-value[2]	q-value[3]
**GENDER**	83									0.8	>0.9
MALE		68 (82%)	18 (86%)	3 (75%)	16 (76%)	12 (75%)	13 (93%)	1 (100%)	5 (83%)		
FEMALE		15 (18%)	3 (14%)	1 (25%)	5 (24%)	4 (25%)	1 (7.1%)	0 (0%)	1 (17%)		
**pSTAGE_binary**	83									0.8	>0.9
IIIa - IV		50 (60%)	14 (67%)	2 (50%)	13 (62%)	9 (56%)	9 (64%)	1 (100%)	2 (33%)		
I & II		33 (40%)	7 (33%)	2 (50%)	8 (38%)	7 (44%)	5 (36%)	0 (0%)	4 (67%)		
**LUACs_Secondary Histological Pattern**	58									>0.9	>0.9
NO SECONDARY		25 (43%)	6 (50%)	1 (50%)	8 (47%)	6 (40%)	2 (29%)	1 (100%)	1 (25%)		
LEPIDIC		2 (3.4%)	0 (0%)	0 (0%)	1 (5.9%)	1 (6.7%)	0 (0%)	0 (0%)	0 (0%)		
ACINAR		17 (29%)	3 (25%)	1 (50%)	5 (29%)	4 (27%)	2 (29%)	0 (0%)	2 (50%)		
PAPILLARY		6 (10%)	1 (8.3%)	0 (0%)	2 (12%)	1 (6.7%)	1 (14%)	0 (0%)	1 (25%)		
MICROPAPILLARY		4 (6.9%)	2 (17%)	0 (0%)	0 (0%)	0 (0%)	2 (29%)	0 (0%)	0 (0%)		
SOLID		4 (6.9%)	0 (0%)	0 (0%)	1 (5.9%)	3 (20%)	0 (0%)	0 (0%)	0 (0%)		
**Tumor_Size**	83									>0.9	>0.9
<=3cm		21 (25%)	6 (29%)	1 (25%)	5 (24%)	3 (19%)	4 (29%)	0 (0%)	2 (33%)		
>3cm & <=5cm		19 (23%)	4 (19%)	1 (25%)	4 (19%)	6 (38%)	3 (21%)	0 (0%)	1 (17%)		

Variable	N	Overall, N = 83[1]	KRAS MUTANT & LKB1 INTACT N = 21[1]	K N = 4[1]	KC N = 21[1]	KL N = 16[1]	KP N = 14[1]	KPL N = 1[1]	L N = 6[1]	p-value[2]	q-value[3]
5cm & <=7cm		27 (33%)	6 (29%)	1 (25%)	7 (33%)	6 (38%)	5 (36%)	1 (100%)	1 (17%)		
>7cm		16 (19%)	5 (24%)	1 (25%)	5 (24%)	1 (6.3%)	2 (14%)	0 (0%)	2 (33%)		
**LUACs_Predominant Histological Pattern**	58									>0.9	>0.9
LEPIDIC		2 (3.4%)	0 (0%)	0 (0%)	1 (5.9%)	1 (6.7%)	0 (0%)	0 (0%)	0 (0%)		
ACINAR		13 (22%)	3 (25%)	0 (0%)	3 (18%)	4 (27%)	3 (43%)	0 (0%)	0 (0%)		
PAPILLARY		5 (8.6%)	1 (8.3%)	0 (0%)	2 (12%)	1 (6.7%)	1 (14%)	0 (0%)	0 (0%)		
MICROPAPILLARY		0 (0%)	0 (0%)	0 (0%)	0 (0%)	0 (0%)	0 (0%)	0 (0%)	0 (0%)		
SOLID		36 (62%)	7 (58%)	2 (100%)	10 (59%)	9 (60%)	3 (43%)	1 (100%)	4 (100%)		
INVASIVE MUCINOUS		2 (3.4%)	1 (8.3%)	0 (0%)	1 (5.9%)	0 (0%)	0 (0%)	0 (0%)	0 (0%)		
COLLOID		0 (0%)	0 (0%)	0 (0%)	0 (0%)	0 (0%)	0 (0%)	0 (0%)	0 (0%)		
ENTERIC		0 (0%)	0 (0%)	0 (0%)	0 (0%)	0 (0%)	0 (0%)	0 (0%)	0 (0%)		
**HISTOTYPE**	83										
ADC		49 (59%)	11 (52%)	2 (50%)	13 (62%)	12 (75%)	6 (43%)	1 (100%)	4 (67%)		
SCC		20 (24%)	8 (38%)	2 (50%)	3 (14%)	0 (0%)	6 (43%)	0 (0%)	1 (17%)		
Pleo ADC		9 (11%)	1 (4.8%)	0 (0%)	4 (19%)	3 (19%)	1 (7.1%)	0 (0%)	0 (0%)		
Pleo SCC		0 (0%)	0 (0%)	0 (0%)	0 (0%)	0 (0%)	0 (0%)	0 (0%)	0 (0%)		
Pleo Spindle Cell		0 (0%)	0 (0%)	0 (0%)	0 (0%)	0 (0%)	0 (0%)	0 (0%)	0 (0%)		
Pleo Large Cell		1 (1.2%)	0 (0%)	0 (0%)	0 (0%)	0 (0%)	0 (0%)	0 (0%)	1 (17%)		

**Variable**	**N**	**Overall,** N = 83[1]	**KRAS MUTANT & LKB1 INTACT** N = 21[1]	**K** N = 4[1]	**KC** N = 21[1]	**KL** N = 16[1]	**KP** N = 14[1]	**KPL** N = 1[1]	**L** N = 6[1]	**p-value**[2]	**q-value**[3]
Large Cell		0 (0%)	0 (0%)	0 (0%)	0 (0%)	0 (0%)	0 (0%)	0 (0%)	0 (0%)		
AdenoSquamous		4 (4.8%)	1 (4.8%)	0 (0%)	1 (4.8%)	1 (6.3%)	1 (7.1%)	0 (0%)	0 (0%)		
**pSTAGE**	83										
IA		1 (1.2%)	0 (0%)	0 (0%)	0 (0%)	0 (0%)	0 (0%)	0 (0%)	1 (17%)		
IB		3 (3.6%)	1 (4.8%)	1 (25%)	0 (0%)	0 (0%)	0 (0%)	0 (0%)	1 (17%)		
IIA		14 (17%)	4 (19%)	1 (25%)	3 (14%)	2 (13%)	3 (21%)	0 (0%)	1 (17%)		
IIB		15 (18%)	2 (9.5%)	0 (0%)	5 (24%)	5 (31%)	2 (14%)	0 (0%)	1 (17%)		
IIIA		49 (59%)	14 (67%)	2 (50%)	13 (62%)	8 (50%)	9 (64%)	1 (100%)	2 (33%)		
IIIB		0 (0%)	0 (0%)	0 (0%)	0 (0%)	0 (0%)	0 (0%)	0 (0%)	0 (0%)		
IV		1 (1.2%)	0 (0%)	0 (0%)	0 (0%)	1 (6.3%)	0 (0%)	0 (0%)	0 (0%)		
**LN_STATUS**	83										
LN0		8 (9.6%)	2 (9.5%)	1 (25%)	1 (4.8%)	0 (0%)	1 (7.1%)	0 (0%)	3 (50%)		
LN1		35 (42%)	8 (38%)	1 (25%)	10 (48%)	7 (44%)	6 (43%)	0 (0%)	3 (50%)		
LN2		11 (13%)	5 (24%)	1 (25%)	1 (4.8%)	1 (6.3%)	3 (21%)	0 (0%)	0 (0%)		
LN3		1 (1.2%)	0 (0%)	0 (0%)	0 (0%)	1 (6.3%)	0 (0%)	0 (0%)	0 (0%)		
LN1 & LN2		28 (34%)	6 (29%)	1 (25%)	9 (43%)	7 (44%)	4 (29%)	1 (100%)	0 (0%)		

[1]n (%)

Variable	N	Overall, N = 83[1]	KRAS MUTANT & LKB1 INTACT N = 21[1]	K N = 4[1]	KC N = 21[1]	KL N = 16[1]	KP N = 14[1]	KPL N = 1[1]	L N = 6[1]	p-value[2]	q-value[3]

[2]Fisher's exact test

[3]False discovery rate correction for multiple testing
