# Supplementary material for: LKB1 Loss Correlates with STING Loss and, in Cooperation with β-Catenin Membranous Loss, Indicates Poor Prognosis in Patients with Operable Non-Small Cell Lung Cancer
Source: Cancers (Basel). 2024 May 10;16(10):1818. doi: 10.3390/cancers16101818 (PMC11120022; doi:10.3390/cancers16101818)
Supplement: Supplementary file 1 [file cancers-16-01818-s001.zip › Supplementary Table S18.pdf]

Table S18 ALL-mOS

Characteristic	Median Survival	p-value[1]
**PDGFRb Tumor**		**<0.001**
0	36.34 (27.47, 50.86)	
1	20.42 (18.33, 24.71)	
**METASTATUS**		**<0.001**
LN META-	46.75 (33.87, —)	
LN META+	21.29 (19.42, 25.79)	
**b-Catenin Membranous**		**0.003**
2-3	36.34 (28.25, 51.61)	
0-1	20.70 (19.38, 25.53)	
**STING**		**0.004**
0	19.68 (16.20, 25.00)	
1	36.53 (26.51, 52.99)	
**p53**		**0.029**
0	34.76 (25.79, 45.47)	
1	19.83 (16.79, 25.53)	
**KRAS**		**0.037**
0	28.25 (23.43, 39.79)	

Characteristic	Median Survival	p-value[1]
1	19.88 (13.24, 34.76)	
**ZEB1 Tumor**		**0.045**
0	37.29 (26.51, 48.36)	
1	21.45 (19.61, 30.98)	
**Cyclin D1**		**0.056**
0	20.09 (17.51, 27.47)	
1	33.23 (23.23, 40.34)	
**PDGFRb Tumor Stroma**		**0.068**
0	48.36 (20.70, 87.92)	
1	24.90 (20.86, 34.37)	
**NEDD9 RNA**		**0.086**
0	20.86 (16.07, 28.25)	
1	36.34 (25.69, 48.36)	
**PDGFRa Tumor Stroma**		0.11
0	39.00 (21.45, 64.36)	
1	24.95 (20.04, 34.37)	
**LKB1 RNA**		0.2
0	23.03 (18.73, 34.76)	

Characteristic	Median Survival	p-value[1]
1	32.66 (22.57, 45.47)	
**CD24**		0.3
0	21.36 (18.73, 36.57)	
1	28.65 (23.43, 43.86)	
**p16**		0.4
0	21.91 (18.73, 40.15)	
1	26.51 (22.47, 38.83)	
**PDGFRa Tumor**		0.4
0	27.47 (20.99, 40.34)	
1	24.02 (19.78, 39.00)	
**VEGFC**		0.5
0	28.65 (24.71, 39.82)	
1	20.91 (16.82, 36.34)	
**PDL1**		0.8
0	25.68 (21.65, 36.57)	
1	25.03 (18.96, 47.15)	
**ZEB1 Tumor Stroma**		0.9
0	23.56 (20.04, 36.30)	

**Characteristic**	**Median Survival**	**p-value**[1]
1	32.00 (21.32, 42.05)	
**BRAF**		>0.9
0	25.68 (20.99, 36.34)	
1	28.11 (21.45, —)	
**LKB1**		>0.9
LOSS	27.47 (20.70, 48.36)	
INTACT	25.66 (20.86, 36.57)	
**pAMPK**		>0.9
0	27.47 (20.70, 48.36)	
1	25.66 (20.86, 36.57)	

[1]Log-rank test
