# Supplementary material for: LKB1 Loss Correlates with STING Loss and, in Cooperation with β-Catenin Membranous Loss, Indicates Poor Prognosis in Patients with Operable Non-Small Cell Lung Cancer
Source: Cancers (Basel). 2024 May 10;16(10):1818. doi: 10.3390/cancers16101818 (PMC11120022; doi:10.3390/cancers16101818)
Supplement: Supplementary file 1 [file cancers-16-01818-s001.zip › Supplementary Table S19.pdf]

Table S19 LKB1 loss _ mOS

Characteristic	Median Survival	p-value[1]
**b-Catenin Membranous**		**<0.001**
2-3	52.99 (34.76, —)	
0-1	20.50 (13.24, 24.90)	
**p53**		0.14
0	34.76 (21.45, 58.15)	
1	18.73 (8.739, —)	
**META STATUS**		0.15
LN META-	56.10 (23.56, —)	
LN META+	22.57 (19.65, 48.00)	
**PDGFRa Tumor**		0.2
0	35.78 (20.99, 78.85)	
1	20.98 (16.56, 58.15)	
**STING**		0.2
0	20.85 (13.24, 41.43)	

Characteristic	Median Survival	p-value[1]
1	48.36 (32.66, —)	
**ZEB1 Tumor Stroma**		0.2
0	20.99 (16.56, 40.34)	
1	44.90 (24.90, —)	
**CD24**		0.3
0	20.50 (16.56, 58.15)	
1	34.76 (20.99, 73.20)	
**VEGFC**		0.3
0	26.18 (20.99, 41.43)	
1	52.99 (17.48, —)	
**KRAS**		0.4
0	27.47 (20.99, 73.20)	
1	27.73 (18.73, —)	
**PDGFRa Tumor Stroma**		0.4
0	20.60 (12.65, —)	
1	34.76 (22.57, 68.76)	

Characteristic	Median Survival	p-value[1]
**BRAF**		0.4
0	31.11 (19.65, 58.15)	
1	23.56 (20.50, —)	
**ZEB1 Tumor**		0.5
0	34.22 (20.70, —)	
1	21.45 (18.73, 73.20)	
**p16**		0.6
0	19.65 (9.232, —)	
1	32.66 (20.99, 58.15)	
**Cyclin**		0.7
0	19.65 (13.24, —)	
1	37.39 (23.56, 58.15)	
**PDGFRb Tumor Stroma**		0.7
0	20.70 (12.19, —)	
1	32.66 (20.99, 58.15)	
**NEDD9 RNA**		0.8

**Characteristic**	**Median Survival**	**p-value**[1]
0	20.99 (16.56, 58.15)	
1	32.66 (22.57, —)	
**PDGFRb Tumor**		0.8
0	24.90 (19.65, 73.20)	
1	36.88 (18.73, —)	
**LKB1 RNA**		0.8
0	23.56 (18.73, 68.76)	
1	32.66 (20.50, —)	
**PD-L1**		>0.9
0	32.66 (20.99, 52.99)	
1	20.52 (13.24, —)	
**Overall**	27.47 (20.70, 48.36)	

[1]Log-rank test
