# Supplementary material for: LKB1 Loss Correlates with STING Loss and, in Cooperation with β-Catenin Membranous Loss, Indicates Poor Prognosis in Patients with Operable Non-Small Cell Lung Cancer
Source: Cancers (Basel). 2024 May 10;16(10):1818. doi: 10.3390/cancers16101818 (PMC11120022; doi:10.3390/cancers16101818)
Supplement: Supplementary file 1 [file cancers-16-01818-s001.zip › Supplementary Table S20.pdf]

Table S20

KL-mOS

Characteristic	Median Survival	p-value[1]
**STING**		**0.011**
0	18.73 (9.232, —)	
1	52.99 (34.76, —)	
**b-catenin Membranous**		**0.069**
2-3	52.99 (34.76, —)	
0-1	20.07 (13.24, —)	
**p16**		0.2
0	19.19 (9.232, —)	
1	34.76 (20.50, —)	
**PDGFRa.Tumor.Stroma**		0.2
0	20.50 (18.73, —)	
1	35.78 (13.24, —)	
**PDGFRb.Tumor.Stroma**		0.2

Characteristic	Median Survival	p-value[1]
0	16.97 (13.24, —)	
1	34.76 (19.65, —)	
**ZEB1.Tumor.Stroma**		0.3
0	19.71 (13.24, —)	
1	35.27 (19.65, —)	
**p53**		0.3
0	34.76 (19.65, —)	
1	18.73 (—, —)	
**CD24**		0.3
0	18.73 (13.24, —)	
1	35.78 (20.70, —)	
**PDGFRa.Tumor**		0.5
0	34.76 (18.73, —)	
1	20.07 (9.232, —)	
**PDL1**		0.5
0	20.70 (18.73, —)	
1	58.15 (13.24, —)	

Characteristic	Median Survival	p-value[1]
**BRAF**		0.5
0	34.76 (18.73, —)	
1	20.50 (—, —)	
**Cyclin**		0.6
0	19.65 (18.73, —)	
1	34.76 (20.50, —)	
**NEDD9.RNA**		0.7
0	20.50 (13.24, —)	
1	40.34 (19.65, —)	
**ZEB1.Tumor**		0.8
0	35.78 (7.951, —)	
1	20.50 (18.73, —)	
**PDGFRb.Tumor**		0.8
0	28.14 (13.24, —)	
1	27.73 (18.73, —)	
**VEGFC**		>0.9
0	34.76 (20.70, —)	

Characteristic	Median Survival	p-value[1]
1	19.61 (9.232, —)	
**LKB1.RNA**		>0.9
0	20.70 (18.73, —)	
1	40.34 (20.50, —)	
**Overall**	27.73 (18.73, —)	
**Overall**	27.73 (18.73, —)	

[1]Log-rank test
