# Supplementary material for: LKB1 Loss Correlates with STING Loss and, in Cooperation with β-Catenin Membranous Loss, Indicates Poor Prognosis in Patients with Operable Non-Small Cell Lung Cancer
Source: Cancers (Basel). 2024 May 10;16(10):1818. doi: 10.3390/cancers16101818 (PMC11120022; doi:10.3390/cancers16101818)
Supplement: Supplementary file 1 [file cancers-16-01818-s001.zip › Supplementary Table S4.pdf]

Table S4 Overall - Clinicopathological Characteristics by Histotype

**Variable**	**N**	**Overall**, N = 248[1]	**LUAC**, N = 110[1]	**LSCC**, N = 119[1]	**Pleo LUAC**, N = 10[1]	**Pleo LSCC**, N = 3[1]	**Pleo Spindle Cell**, N = 1[1]	**Pleo Large Cell**, N = 1[1]	**Large Cell**, N = 1[1]	**AdenoSquamous**, N = 3[1]	**p-value**[2]	**q-value**[3]
**GENDER**	248										0.002	0.011
MALE		220 (89%)	88 (80%)	115 (97%)	9 (90%)	3 (100%)	1 (100%)	1 (100%)	1 (100%)	2 (67%)		
FEMALE		28 (11%)	22 (20%)	4 (3.4%)	1 (10%)	0 (0%)	0 (0%)	0 (0%)	0 (0%)	1 (33%)		
**GRADE**	248										0.021	0.064
G1		14 (5.6%)	12 (11%)	2 (1.7%)	0 (0%)	0 (0%)	0 (0%)	0 (0%)	0 (0%)	0 (0%)		
G2		83 (33%)	35 (32%)	46 (39%)	0 (0%)	0 (0%)	0 (0%)	0 (0%)	0 (0%)	2 (67%)		
G3		151 (61%)	63 (57%)	71 (60%)	10 (100%)	3 (100%)	1 (100%)	1 (100%)	1 (100%)	1 (33%)		
**AGE AT DIAGNOSIS**	248										0.033	0.073
<70		181 (73%)	90 (82%)	77 (65%)	7 (70%)	3 (100%)	1 (100%)	1 (100%)	1 (100%)	1 (33%)		
>=70		67 (27%)	20 (18%)	42 (35%)	3 (30%)	0 (0%)	0 (0%)	0 (0%)	0 (0%)	2 (67%)		
**Tumor Size**	248										0.064	0.12
<=3cm		75 (30%)	43 (39%)	27 (23%)	3 (30%)	2 (67%)	0 (0%)	0 (0%)	0 (0%)	0 (0%)		
>3cm & <=5cm		79 (32%)	36 (33%)	37 (31%)	2 (20%)	1 (33%)	0 (0%)	1 (100%)	0 (0%)	2 (67%)		

Variable	N	**Overall**, N = 248[1]	**LUAC**, N = 110[1]	**LSCC**, N = 119[1]	**Pleo LUAC**, N = 10[1]	**Pleo LSCC**, N = 3[1]	**Pleo Spindle Cell**, N = 1[1]	**Pleo Large Cell**, N = 1[1]	**Large Cell**, N = 1[1]	**AdenoSquamous**, N = 3[1]	**p-value**[2]	**q-value**[3]
5cm & <=7cm		48 (19%)	14 (13%)	29 (24%)	3 (30%)	0 (0%)	0 (0%)	0 (0%)	1 (100%)	1 (33%)		
>7cm		46 (19%)	17 (15%)	26 (22%)	2 (20%)	0 (0%)	1 (100%)	0 (0%)	0 (0%)	0 (0%)		
**META STATUS**	248										0.3	0.4
LN META-		60 (24%)	28 (25%)	25 (21%)	3 (30%)	2 (67%)	0 (0%)	1 (100%)	0 (0%)	1 (33%)		
LN META+		188 (76%)	82 (75%)	94 (79%)	7 (70%)	1 (33%)	1 (100%)	0 (0%)	1 (100%)	2 (67%)		
**LN STATUS**	248											
LN0		60 (24%)	28 (25%)	25 (21%)	3 (30%)	2 (67%)	0 (0%)	1 (100%)	0 (0%)	1 (33%)		
LN1		106 (43%)	38 (35%)	61 (51%)	4 (40%)	1 (33%)	0 (0%)	0 (0%)	1 (100%)	1 (33%)		0.5
LN2		21 (8.5%)	13 (12%)	7 (5.9%)	0 (0%)	0 (0%)	1 (100%)	0 (0%)	0 (0%)	0 (0%)		
LN3		2 (0.8%)	2 (1.8%)	0 (0%)	0 (0%)	0 (0%)	0 (0%)	0 (0%)	0 (0%)	0 (0%)		
LN1 & LN2		59 (24%)	29 (26%)	26 (22%)	3 (30%)	0 (0%)	0 (0%)	0 (0%)	0 (0%)	1 (33%)		
**LUACs_Predominant Histological Pattern**	120										0.4	0.5
LEPIDIC		5 (4.2%)	5 (4.5%)	0 (NA%)	0 (0%)	0 (NA%)	0 (NA%)	0 (NA%)	0 (NA%)	0 (NA%)		
ACINAR		30 (25%)	30 (27%)	0 (NA%)	0 (0%)	0 (NA%)	0 (NA%)	0 (NA%)	0 (NA%)	0 (NA%)		

**Variable**	**N**	**Overall**, N = 248[1]	**LUAC**, N = 110[1]	**LSCC**, N = 119[1]	**Pleo LUAC**, N = 10[1]	**Pleo LSCC**, N = 3[1]	**Pleo Spindle Cell**, N = 1[1]	**Pleo Large Cell**, N = 1[1]	**Large Cell**, N = 1[1]	**AdenoSquamous**, N = 3[1]	**p-value**[2]	**q-value**[3]
PAPILLARY		12 (10%)	11 (10%)	0 (NA%)	1 (10%)	0 (NA%)	0 (NA%)	0 (NA%)	0 (NA%)	0 (NA%)		
MICROPAPILLARY		1 (0.8%)	1 (0.9%)	0 (NA%)	0 (0%)	0 (NA%)	0 (NA%)	0 (NA%)	0 (NA%)	0 (NA%)		
SOLID		62 (52%)	53 (48%)	0 (NA%)	9 (90%)	0 (NA%)	0 (NA%)	0 (NA%)	0 (NA%)	0 (NA%)		
INVASIVE MUCINOUS		3 (2.5%)	3 (2.7%)	0 (NA%)	0 (0%)	0 (NA%)	0 (NA%)	0 (NA%)	0 (NA%)	0 (NA%)		
COLLOID		3 (2.5%)	3 (2.7%)	0 (NA%)	0 (0%)	0 (NA%)	0 (NA%)	0 (NA%)	0 (NA%)	0 (NA%)		
ENTERIC		4 (3.3%)	4 (3.6%)	0 (NA%)	0 (0%)	0 (NA%)	0 (NA%)	0 (NA%)	0 (NA%)	0 (NA%)		
**LUACs_Secondary Histological Pattern**	120										0.7	0.8
NO SECONDARY		42 (35%)	38 (35%)	0 (NA%)	4 (40%)	0 (NA%)	0 (NA%)	0 (NA%)	0 (NA%)	0 (NA%)		
LEPIDIC		6 (5.0%)	6 (5.5%)	0 (NA%)	0 (0%)	0 (NA%)	0 (NA%)	0 (NA%)	0 (NA%)	0 (NA%)		
ACINAR		31 (26%)	27 (25%)	0 (NA%)	4 (40%)	0 (NA%)	0 (NA%)	0 (NA%)	0 (NA%)	0 (NA%)		
PAPILLARY		17 (14%)	15 (14%)	0 (NA%)	2 (20%)	0 (NA%)	0 (NA%)	0 (NA%)	0 (NA%)	0 (NA%)		
MICROPAPILLARY		15 (13%)	15 (14%)	0 (NA%)	0 (0%)	0 (NA%)	0 (NA%)	0 (NA%)	0 (NA%)	0 (NA%)		
SOLID		9 (7.5%)	9 (8.2%)	0 (NA%)	0 (0%)	0 (NA%)	0 (NA%)	0 (NA%)	0 (NA%)	0 (NA%)		

**Variable**	**N**	**Overall**, N = 248[1]	**LUAC**, N = 110[1]	**LSCC**, N = 119[1]	**Pleo LUAC**, N = 10[1]	**Pleo LSCC**, N = 3[1]	**Pleo Spindle Cell**, N = 1[1]	**Pleo Large Cell**, N = 1[1]	**Large Cell**, N = 1[1]	**AdenoSquamous**, N = 3[1]	**p-value**[2]	**q-value**[3]
**p-STAGE**	248										0.8	0.8
IIIa - IV		123 (50%)	57 (52%)	56 (47%)	6 (60%)	1 (33%)	1 (100%)	0 (0%)	1 (100%)	1 (33%)		
I & II		125 (50%)	53 (48%)	63 (53%)	4 (40%)	2 (67%)	0 (0%)	1 (100%)	0 (0%)	2 (67%)		
IIIA		121 (49%)	55 (50%)	56 (47%)	6 (60%)	1 (33%)	1 (100%)	0 (0%)	1 (100%)	1 (33%)		
IIIB		1 (0.4%)	1 (0.9%)	0 (0%)	0 (0%)	0 (0%)	0 (0%)	0 (0%)	0 (0%)	0 (0%)		
IV		1 (0.4%)	1 (0.9%)	0 (0%)	0 (0%)	0 (0%)	0 (0%)	0 (0%)	0 (0%)	0 (0%)		

**Variable**	**N**	**Overall**, N = 248[1]	**LUAC**, N = 110[1]	**LSCC**, N = 119[1]	**Pleo LUAC**, N = 10[1]	**Pleo LSCC**, N = 3[1]	**Pleo Spindle Cell**, N = 1[1]	**Pleo Large Cell**, N = 1[1]	**Large Cell**, N = 1[1]	**AdenoSquamous**, N = 3[1]	**p-value**[2]	**q-value**[3]

[1]n (%)

[2]Fisher's exact test

[3]False discovery rate correction for multiple testing
