# Supplementary material for: LKB1 Loss Correlates with STING Loss and, in Cooperation with β-Catenin Membranous Loss, Indicates Poor Prognosis in Patients with Operable Non-Small Cell Lung Cancer
Source: Cancers (Basel). 2024 May 10;16(10):1818. doi: 10.3390/cancers16101818 (PMC11120022; doi:10.3390/cancers16101818)
Supplement: Supplementary file 1 [file cancers-16-01818-s001.zip › Supplementary Table S5.pdf]

Table S5 Overall - Laboratory

Analysis by Histotype

Variable	N	Overall, N = 248[1]	LUAC, N = 110[1]	LSCC, N = 119[1]	Pleo LUAC, N = 10[1]	Pleo LSCC, N = 3[1]	Pleo Spindle Cell, N = 1[1]	Pleo Large Cell, N = 1[1]	Large Cell, N = 1[1]	AdenoSquamous, N = 3[1]	p-value[2]	q-value[3]
**CD24**	248										<0.001	<0.001
0		113 (46%)	24 (22%)	77 (65%)	7 (70%)	3 (100%)	1 (100%)	0 (0%)	0 (0%)	1 (33%)		
1		135 (54%)	86 (78%)	42 (35%)	3 (30%)	0 (0%)	0 (0%)	1 (100%)	1 (100%)	2 (67%)		
**LKB1_ TUMOR**	248										<0.001	<0.001
LOSS		51 (21%)	36 (33%)	7 (5.9%)	5 (50%)	0 (0%)	0 (0%)	1 (100%)	1 (100%)	1 (33%)		
INTACT		197 (79%)	74 (67%)	112 (94%)	5 (50%)	3 (100%)	1 (100%)	0 (0%)	0 (0%)	2 (67%)		
**pAMPK_TUMOR**	248										<0.001	<0.001
0		51 (21%)	36 (33%)	7 (5.9%)	5 (50%)	0 (0%)	0 (0%)	1 (100%)	1 (100%)	1 (33%)		
1		197 (79%)	74 (67%)	112 (94%)	5 (50%)	3 (100%)	1 (100%)	0 (0%)	0 (0%)	2 (67%)		
**PDGFRa_TUMOR_STROMA**	248										<0.001	<0.001

Variable	N	Overall, N = 248[1]	LUAC, N = 110[1]	LSCC, N = 119[1]	Pleo LUAC, N = 10[1]	Pleo LSCC, N = 3[1]	Pleo Spindle Cell, N = 1[1]	Pleo Large Cell, N = 1[1]	Large Cell, N = 1[1]	AdenoSquamous, N = 3[1]	p-value[2]	q-value[3]
0		65 (26%)	40 (36%)	15 (13%)	6 (60%)	2 (67%)	1 (100%)	1 (100%)	0 (0%)	0 (0%)		
1		183 (74%)	70 (64%)	104 (87%)	4 (40%)	1 (33%)	0 (0%)	0 (0%)	1 (100%)	3 (100%)		
**ZEB1_TUMOR**	248										<0.001	<0.001
0		90 (36%)	57 (52%)	29 (24%)	1 (10%)	2 (67%)	0 (0%)	1 (100%)	0 (0%)	0 (0%)		
1		158 (64%)	53 (48%)	90 (76%)	9 (90%)	1 (33%)	1 (100%)	0 (0%)	1 (100%)	3 (100%)		
**KL**	248										<0.001	<0.001
NO KL		232 (94%)	98 (89%)	119 (100%)	7 (70%)	3 (100%)	1 (100%)	1 (100%)	1 (100%)	2 (67%)		
KL		16 (6.5%)	12 (11%)	0 (0%)	3 (30%)	0 (0%)	0 (0%)	0 (0%)	0 (0%)	1 (33%)		
**PD-L1_TUMOR_SCORE_ TPS**	248										<0.001	0.002
0		159 (64%)	81 (74%)	72 (61%)	2 (20%)	0 (0%)	0 (0%)	1 (100%)	1 (100%)	2 (67%)		
1		89 (36%)	29 (26%)	47 (39%)	8 (80%)	3 (100%)	1 (100%)	0 (0%)	0 (0%)	1 (33%)		

Variable	N	Overall, N = 248[1]	LUAC, N = 110[1]	LSCC, N = 119[1]	Pleo LUAC, N = 10[1]	Pleo LSCC, N = 3[1]	Pleo Spindle Cell, N = 1[1]	Pleo Large Cell, N = 1[1]	Large Cell, N = 1[1]	AdenoSquamous, N = 3[1]	p-value[2]	q-value[3]
**KRAS**	248										0.001	0.004
0		211 (85%)	87 (79%)	111 (93%)	6 (60%)	3 (100%)	1 (100%)	1 (100%)	1 (100%)	1 (33%)		
1		37 (15%)	23 (21%)	8 (6.7%)	4 (40%)	0 (0%)	0 (0%)	0 (0%)	0 (0%)	2 (67%)		
**ZEB1_TUMOR STROMA**	248										0.002	0.005
0		121 (49%)	65 (59%)	43 (36%)	7 (70%)	1 (33%)	1 (100%)	1 (100%)	1 (100%)	2 (67%)		
1		127 (51%)	45 (41%)	76 (64%)	3 (30%)	2 (67%)	0 (0%)	0 (0%)	0 (0%)	1 (33%)		
**KC**	248										0.002	0.005
NO KC		227 (92%)	97 (88%)	116 (97%)	6 (60%)	3 (100%)	1 (100%)	1 (100%)	1 (100%)	2 (67%)		
KC		21 (8.5%)	13 (12%)	3 (2.5%)	4 (40%)	0 (0%)	0 (0%)	0 (0%)	0 (0%)	1 (33%)		
**bCatenin_TUMOR_MEMBRANOUS**	248										**0.005**	0.013
2-3		128 (52%)	70 (64%)	51 (43%)	3 (30%)	1 (33%)	0 (0%)	1 (100%)	0 (0%)	2 (67%)		
0-1		120 (48%)	40 (36%)	68 (57%)	7 (70%)	2 (67%)	1 (100%)	0 (0%)	1 (100%)	1 (33%)		

Variable	N	Overall, N = 248[1]	LUAC, N = 110[1]	LSCC, N = 119[1]	Pleo LUAC, N = 10[1]	Pleo LSCC, N = 3[1]	Pleo Spindle Cell, N = 1[1]	Pleo Large Cell, N = 1[1]	Large Cell, N = 1[1]	AdenoSquamous, N = 3[1]	p-value[2]	q-value[3]
**PDGFRb_TUMOR_STROMA**	248										0.029	0.062
0		43 (17%)	23 (21%)	14 (12%)	3 (30%)	1 (33%)	1 (100%)	1 (100%)	0 (0%)	0 (0%)		
1		205 (83%)	87 (79%)	105 (88%)	7 (70%)	2 (67%)	0 (0%)	0 (0%)	1 (100%)	3 (100%)		
**LKB1_RNA_TUMOR**	248										0.045	0.089
0		124 (50%)	64 (58%)	48 (40%)	6 (60%)	2 (67%)	1 (100%)	1 (100%)	1 (100%)	1 (33%)		
1		124 (50%)	46 (42%)	71 (60%)	4 (40%)	1 (33%)	0 (0%)	0 (0%)	0 (0%)	2 (67%)		
**L**	248										0.051	0.092
NO L		242 (98%)	106 (96%)	118 (99%)	10 (100%)	3 (100%)	1 (100%)	0 (0%)	1 (100%)	3 (100%)		
L		6 (2.4%)	4 (3.6%)	1 (0.8%)	0 (0%)	0 (0%)	0 (0%)	1 (100%)	0 (0%)	0 (0%)		
**p53**	248										0.053	0.092

Variable	N	Overall, N = 248[1]	LUAC, N = 110[1]	LSCC, N = 119[1]	Pleo LUAC, N = 10[1]	Pleo LSCC, N = 3[1]	Pleo Spindle Cell, N = 1[1]	Pleo Large Cell, N = 1[1]	Large Cell, N = 1[1]	AdenoSquamous, N = 3[1]	p-value[2]	q-value[3]
0		147 (59%)	73 (66%)	66 (55%)	6 (60%)	0 (0%)	0 (0%)	1 (100%)	0 (0%)	1 (33%)		
1		101 (41%)	37 (34%)	53 (45%)	4 (40%)	3 (100%)	1 (100%)	0 (0%)	1 (100%)	2 (67%)		
**PDGFRb_TUMOR**	248										0.2	0.3
0		124 (50%)	55 (50%)	57 (48%)	5 (50%)	3 (100%)	0 (0%)	1 (100%)	0 (0%)	3 (100%)		
1		124 (50%)	55 (50%)	62 (52%)	5 (50%)	0 (0%)	1 (100%)	0 (0%)	1 (100%)	0 (0%)		
**Cyclin-D1**	248										0.3	0.4
0		66 (27%)	26 (24%)	33 (28%)	4 (40%)	0 (0%)	0 (0%)	0 (0%)	1 (100%)	2 (67%)		
1		182 (73%)	84 (76%)	86 (72%)	6 (60%)	3 (100%)	1 (100%)	1 (100%)	0 (0%)	1 (33%)		
**NEDD9_TUMOR**	248										0.3	0.4
0		123 (50%)	51 (46%)	60 (50%)	8 (80%)	1 (33%)	0 (0%)	1 (100%)	1 (100%)	1 (33%)		
1		125 (50%)	59 (54%)	59 (50%)	2 (20%)	2 (67%)	1 (100%)	0 (0%)	0 (0%)	2 (67%)		

Variable	N	Overall, N = 248[1]	LUAC, N = 110[1]	LSCC, N = 119[1]	Pleo LUAC, N = 10[1]	Pleo LSCC, N = 3[1]	Pleo Spindle Cell, N = 1[1]	Pleo Large Cell, N = 1[1]	Large Cell, N = 1[1]	AdenoSquamous, N = 3[1]	p-value[2]	q-value[3]
**VEGFC**	248										0.3	0.4
0		134 (54%)	56 (51%)	65 (55%)	5 (50%)	3 (100%)	0 (0%)	1 (100%)	1 (100%)	3 (100%)		
1		114 (46%)	54 (49%)	54 (45%)	5 (50%)	0 (0%)	1 (100%)	0 (0%)	0 (0%)	0 (0%)		
**PDGFRa_TUMOR**	248										0.3	0.4
0		116 (47%)	56 (51%)	50 (42%)	3 (30%)	2 (67%)	1 (100%)	1 (100%)	1 (100%)	2 (67%)		
1		132 (53%)	54 (49%)	69 (58%)	7 (70%)	1 (33%)	0 (0%)	0 (0%)	0 (0%)	1 (33%)		
**p16**	248										0.4	0.4
0		71 (29%)	38 (35%)	29 (24%)	2 (20%)	0 (0%)	0 (0%)	1 (100%)	0 (0%)	1 (33%)		
1		177 (71%)	72 (65%)	90 (76%)	8 (80%)	3 (100%)	1 (100%)	0 (0%)	1 (100%)	2 (67%)		
**KP**	248										0.4	0.4
NO KP		234 (94%)	104 (95%)	113 (95%)	9 (90%)	3 (100%)	1 (100%)	1 (100%)	1 (100%)	2 (67%)		

Variable	N	Overall, N = 248[1]	LUAC, N = 110[1]	LSCC, N = 119[1]	Pleo LUAC, N = 10[1]	Pleo LSCC, N = 3[1]	Pleo Spindle Cell, N = 1[1]	Pleo Large Cell, N = 1[1]	Large Cell, N = 1[1]	AdenoSquamous, N = 3[1]	p-value[2]	q-value[3]
KP		14 (5.6%)	6 (5.5%)	6 (5.0%)	1 (10%)	0 (0%)	0 (0%)	0 (0%)	0 (0%)	1 (33%)		
**KPL**	248										0.5	0.6
NO KPL		247 (100%)	109 (99%)	119 (100%)	10 (100%)	3 (100%)	1 (100%)	1 (100%)	1 (100%)	3 (100%)		
KPL		1 (0.4%)	1 (0.9%)	0 (0%)	0 (0%)	0 (0%)	0 (0%)	0 (0%)	0 (0%)	0 (0%)		
**STING_TUMOR**	248										0.6	0.7
0		123 (50%)	49 (45%)	63 (53%)	4 (40%)	2 (67%)	1 (100%)	1 (100%)	1 (100%)	2 (67%)		
1		125 (50%)	61 (55%)	56 (47%)	6 (60%)	1 (33%)	0 (0%)	0 (0%)	0 (0%)	1 (33%)		
**BRAF_TUMOR**	244										>0.9	>0.9
0		224 (92%)	99 (91%)	107 (92%)	9 (90%)	3 (100%)	1 (100%)	1 (100%)	1 (100%)	3 (100%)		
1		20 (8.2%)	10 (9.2%)	9 (7.8%)	1 (10%)	0 (0%)	0 (0%)	0 (0%)	0 (0%)	0 (0%)		
**K**	248										>0.9	>0.9

**Variable**	**N**	**Overall**, N = 248[1]	**LUAC**, N = 110[1]	**LSCC**, N = 119[1]	**Pleo LUAC**, N = 10[1]	**Pleo LSCC**, N = 3[1]	**Pleo Spindle Cell**, N = 1[1]	**Pleo Large Cell**, N = 1[1]	**Large Cell**, N = 1[1]	**AdenoSquamous**, N = 3[1]	**p-value**[2]	**q-value**[3]
NO K		244 (98%)	108 (98%)	117 (98%)	10 (100%)	3 (100%)	1 (100%)	1 (100%)	1 (100%)	3 (100%)		
K		4 (1.6%)	2 (1.8%)	2 (1.7%)	0 (0%)	0 (0%)	0 (0%)	0 (0%)	0 (0%)	0 (0%)		

[1]n (%)

[2]Fisher's exact test

[3]False discovery rate correction for multiple testing
