# Supplementary material for: LKB1 Loss Correlates with STING Loss and, in Cooperation with β-Catenin Membranous Loss, Indicates Poor Prognosis in Patients with Operable Non-Small Cell Lung Cancer
Source: Cancers (Basel). 2024 May 10;16(10):1818. doi: 10.3390/cancers16101818 (PMC11120022; doi:10.3390/cancers16101818)
Supplement: Supplementary file 1 [file cancers-16-01818-s001.zip › Supplementary Table S7.pdf]

Table S7 - Laboratory Characteristics

Non-Pleomorphic LUACS vs Pleomorphic LUACS

**Variable**	**N**	**Overall**, N = 120[1]	**LUAC**, N = 110[1]	**Pleo LUAC**, N = 10[1]	**p-value**[2]	**q-value**[3]
**PD-L1_TUMOR- TPS**	120				**0.001**	0.032
0		83 (69%)	81 (74%)	2 (20%)		
1		37 (31%)	29 (26%)	**8 (80%)**		
**CD24**	120				**0.003**	0.037
0		31 (26%)	24 (22%)	7 (70%)		
1		89 (74%)	86 (78%)	3 (30%)		
**ZEB1_TUMOR**	120				**0.017**	0.15
0		58 (48%)	57 (52%)	1 (10%)		
1		62 (52%)	53 (48%)	**9 (90%)**		
**KC**	120				**0.034**	0.2
NO KC		103 (86%)	97 (88%)	6 (60%)		
KC		17 (14%)	13 (12%)	**4 (40%)**		
**b-Catenin_TUMOR_MEMBRANOUS**	120				**0.024**	0.2
2-3		73 (61%)	70 (64%)	3 (30%)		

**Variable**	**N**	**Overall**, N = 120[1]	**LUAC**, N = 110[1]	**Pleo LUAC**, N = 10[1]	**p-value**[2]	**q-value**[3]
0-1		47 (39%)	40 (36%)	**7 (70%)**		
**NEDD9_TUMOR**	120				0.052	0.2
0		59 (49%)	51 (46%)	8 (80%)		
1		61 (51%)	59 (54%)	2 (20%)		
**KL**	120				0.11	0.4
NO KL		105 (88%)	98 (89%)	7 (70%)		
KL		15 (13%)	12 (11%)	3 (30%)		
**PDGFRa_TUMOR_STROMA**	120				0.2	0.6
0		46 (38%)	40 (36%)	6 (60%)		
1		74 (62%)	70 (64%)	4 (40%)		
**KRAS**	120				0.2	0.6
0		93 (78%)	87 (79%)	6 (60%)		
1		27 (23%)	23 (21%)	4 (40%)		
**Cyclin-D1**	120				0.3	0.6
0		30 (25%)	26 (24%)	4 (40%)		
1		90 (75%)	84 (76%)	6 (60%)		

Variable	N	Overall, N = 120[1]	LUAC, N = 110[1]	Pleo LUAC, N = 10[1]	p-value[2]	q-value[3]
**LKB1_TUMOR**	120				0.3	0.6
LOSS		41 (34%)	36 (33%)	5 (50%)		
INTACT		79 (66%)	74 (67%)	5 (50%)		
**pAMPK_TUMOR**	120				0.3	0.6
0		41 (34%)	36 (33%)	5 (50%)		
1		79 (66%)	74 (67%)	5 (50%)		
**PDGFRa_TUMOR**	120				0.3	0.6
0		59 (49%)	56 (51%)	3 (30%)		
1		61 (51%)	54 (49%)	7 (70%)		
**PDGFRb_TUMOR_STROMA**	120				0.5	0.8
0		26 (22%)	23 (21%)	3 (30%)		
1		94 (78%)	87 (79%)	7 (70%)		
**KP**	120				0.5	0.8
NO KP		113 (94%)	104 (95%)	9 (90%)		
KP		7 (5.8%)	6 (5.5%)	1 (10%)		
**p16**	120				0.5	0.8

**Variable**	**N**	**Overall**, N = 120[1]	**LUAC**, N = 110[1]	**Pleo LUAC**, N = 10[1]	**p-value**[2]	**q-value**[3]
0		40 (33%)	38 (35%)	2 (20%)		
1		80 (67%)	72 (65%)	8 (80%)		
**p53**	120				0.7	>0.9
0		79 (66%)	73 (66%)	6 (60%)		
1		41 (34%)	37 (34%)	4 (40%)		
**ZEB1_TUMOR_STROMA**	120				0.7	>0.9
0		72 (60%)	65 (59%)	7 (70%)		
1		48 (40%)	45 (41%)	3 (30%)		
**BRAF_TUMOR**	119				>0.9	>0.9
0		108 (91%)	99 (91%)	9 (90%)		
1		11 (9.2%)	10 (9.2%)	1 (10%)		
**VEGFC**	120				>0.9	>0.9
0		61 (51%)	56 (51%)	5 (50%)		
1		59 (49%)	54 (49%)	5 (50%)		
**STING_TUMOR**	120				>0.9	>0.9
0		53 (44%)	49 (45%)	4 (40%)		

**Variable**	**N**	**Overall**, N = 120[1]	**LUAC**, N = 110[1]	**Pleo LUAC**, N = 10[1]	**p-value**[2]	**q-value**[3]
1		67 (56%)	61 (55%)	6 (60%)		
**PDGFRb_TUMOR**	120				>0.9	>0.9
0		60 (50%)	55 (50%)	5 (50%)		
1		60 (50%)	55 (50%)	5 (50%)		
**LKB1_RNA_TUMOR**	120				>0.9	>0.9
0		70 (58%)	64 (58%)	6 (60%)		
1		50 (42%)	46 (42%)	4 (40%)		
**K**	120				>0.9	>0.9
NO K		118 (98%)	108 (98%)	10 (100%)		
K		2 (1.7%)	2 (1.8%)	0 (0%)		
**KPL**	120				>0.9	>0.9
NO KPL		119 (99%)	109 (99%)	10 (100%)		
KPL		1 (0.8%)	1 (0.9%)	0 (0%)		
**L**	120				>0.9	>0.9
NO L		116 (97%)	106 (96%)	10 (100%)		
L		4 (3.3%)	4 (3.6%)	0 (0%)		

**Variable**	**N**	**Overall**, N = 120[1]	**LUAC**, N = 110[1]	**Pleo LUAC**, N = 10[1]	**p-value**[2]	**q-value**[3]

[1]n (%)

[2]Fisher's exact test; Pearson's Chi-squared test

[3]False discovery rate correction for multiple testing
