# Supplementary material for: LKB1 Loss Correlates with STING Loss and, in Cooperation with β-Catenin Membranous Loss, Indicates Poor Prognosis in Patients with Operable Non-Small Cell Lung Cancer
Source: Cancers (Basel). 2024 May 10;16(10):1818. doi: 10.3390/cancers16101818 (PMC11120022; doi:10.3390/cancers16101818)
Supplement: Supplementary file 1 [file cancers-16-01818-s001.zip › Supplementary Table S8.pdf]

Table S8 Non-Pleomorphic LUACs vs Pleomorphic LUACs - Laboratory Characteristics

Variable	N	Overall, N = 122[1]	SCC, N = 119[1]	Pleo SCC, N = 3[1]	p-value[2]	q-value[3]
**PDGFRa_TUMOR_STROMA**	122				0.05	0.8
0		17 (14%)	15 (13%)	2 (67%)		
1		105 (86%)	104 (87%)	1 (33%)		
**PD-L1_TUMOR_SCORE- TPS**	122				**0.05**	0.8
0		72 (59%)	72 (61%)	0 (0%)		
1		50 (41%)	47 (39%)	3 (100%)		
**p53**	122				0.074	0.8
0		66 (54%)	66 (55%)	0 (0%)		
1		56 (46%)	53 (45%)	3 (100%)		
**PDGFRb_TUMOR**	122				0.12	0.8
0		60 (49%)	57 (48%)	3 (100%)		
1		62 (51%)	62 (52%)	0 (0%)		
**ZEB1_TUMOR**	122				0.2	0.8
0		31 (25%)	29 (24%)	2 (67%)		
1		91 (75%)	90 (76%)	1 (33%)		
**VEGFC**	122				0.3	>0.9
0		68 (56%)	65 (55%)	3 (100%)		
1		54 (44%)	54 (45%)	0 (0%)		
**PDGFRb_TUMOR_STROMA**	122				0.3	>0.9

**Variable**	**N**	**Overall**, N = 122[1]	**SCC**, N = 119[1]	**Pleo SCC**, N = 3[1]	**p-value**[2]	**q-value**[3]
0		15 (12%)	14 (12%)	1 (33%)		
1		107 (88%)	105 (88%)	2 (67%)		
**CD24**	122				0.6	>0.9
0		80 (66%)	77 (65%)	3 (100%)		
1		42 (34%)	42 (35%)	0 (0%)		
**Cyclin_D1**	122				0.6	>0.9
0		33 (27%)	33 (28%)	0 (0%)		
1		89 (73%)	86 (72%)	3 (100%)		
**LKB1_RNA_TUMOR**	122				0.6	>0.9
0		50 (41%)	48 (40%)	2 (67%)		
1		72 (59%)	71 (60%)	1 (33%)		
**PDGFRa_TUMOR**	122				0.6	>0.9
0		52 (43%)	50 (42%)	2 (67%)		
1		70 (57%)	69 (58%)	1 (33%)		
**ZEB1_TUMOR_STROMA**	122				>0.9	>0.9
0		44 (36%)	43 (36%)	1 (33%)		
1		78 (64%)	76 (64%)	2 (67%)		
**LKB1_TUMOR**	122				>0.9	>0.9
LOSS		7 (5.7%)	7 (5.9%)	0 (0%)		
INTACT		115 (94%)	112 (94%)	3 (100%)		
**p16**	122				>0.9	>0.9

**Variable**	**N**	**Overall**, N = 122[1]	**SCC**, N = 119[1]	**Pleo SCC**, N = 3[1]	**p-value**[2]	**q-value**[3]
0		29 (24%)	29 (24%)	0 (0%)		
1		93 (76%)	90 (76%)	3 (100%)		
**KRAS_TUMOR**	122				>0.9	>0.9
0		114 (93%)	111 (93%)	3 (100%)		
1		8 (6.6%)	8 (6.7%)	0 (0%)		
**BRAF_ TUMOR**	119				>0.9	>0.9
0		110 (92%)	107 (92%)	3 (100%)		
1		9 (7.6%)	9 (7.8%)	0 (0%)		
**pAMPK_TUMOR**	122				>0.9	>0.9
0		7 (5.7%)	7 (5.9%)	0 (0%)		
1		115 (94%)	112 (94%)	3 (100%)		
**STING_TUMOR**	122				>0.9	>0.9
0		65 (53%)	63 (53%)	2 (67%)		
1		57 (47%)	56 (47%)	1 (33%)		
**b-Catenin_TUMOR_MEMBRANOUS**	122				>0.9	>0.9
2-3		52 (43%)	51 (43%)	1 (33%)		
0-1		70 (57%)	68 (57%)	2 (67%)		
**NEDD9_TUMOR**	122				>0.9	>0.9
0		61 (50%)	60 (50%)	1 (33%)		
1		61 (50%)	59 (50%)	2 (67%)		
**K**	122				>0.9	>0.9

Variable	N	**Overall**, N = 122[1]	**SCC**, N = 119[1]	**Pleo SCC**, N = 3[1]	p-value[2]	q-value[3]
NO K		120 (98%)	117 (98%)	3 (100%)		
K		2 (1.6%)	2 (1.7%)	0 (0%)		
**KPL**	122				>0.9	>0.9
NO KPL		122 (100%)	119 (100%)	3 (100%)		
KPL		0 (0%)	0 (0%)	0 (0%)		
**KL**	122				>0.9	>0.9
NO KL		122 (100%)	119 (100%)	3 (100%)		
KL		0 (0%)	0 (0%)	0 (0%)		
**KC**	122				>0.9	>0.9
NO KC		119 (98%)	116 (97%)	3 (100%)		
KC		3 (2.5%)	3 (2.5%)	0 (0%)		
**KP**	122				>0.9	>0.9
NO KP		116 (95%)	113 (95%)	3 (100%)		
KP		6 (4.9%)	6 (5.0%)	0 (0%)		
**L**	122				>0.9	>0.9
NO L		121 (99%)	118 (99%)	3 (100%)		
L		1 (0.8%)	1 (0.8%)	0 (0%)		

[1]n (%)

[2]Fisher's exact test

[3]False discovery rate correction for multiple testing
