# Supplementary material for: LKB1 Loss Correlates with STING Loss and, in Cooperation with β-Catenin Membranous Loss, Indicates Poor Prognosis in Patients with Operable Non-Small Cell Lung Cancer
Source: Cancers (Basel). 2024 May 10;16(10):1818. doi: 10.3390/cancers16101818 (PMC11120022; doi:10.3390/cancers16101818)
Supplement: Supplementary file 1 [file cancers-16-01818-s001.zip › Supplementary Table S9.pdf]

Table S9 Overall - LKB1 loss vs LKB1 intact - Clinicopathological Characteristics

Variable	N	Overall, N = 248[1]	LKB1 LOSS, N = 51[1]	LKB1 INTACT, N = 197[1]	p-value[2]	q-value[3]
**HISTOTYPE**	248				**<0.001**	<0.001
LUAC		110 (44%)	**36 (71%)**	**74 (38%)**		
LSCC		119 (48%)	**7 (14%)**	112 (57%)		
Pleo-LUAC		10 (4.0%)	**5 (9.8%)**	**5 (2.5%)**		
Pleo-LSCC		3 (1.2%)	0 (0%)	3 (1.5%)		
Pleo-Spindle Cell		1 (0.4%)	1 (0.5%)	0 (0%)		
Pleo-Large Cell		1 (0.4%)	1 (2.0%)	0 (0%)		
Large Cell		1 (0.4%)	1 (2.0%)	0 (0%)		
Adeno-Squamous		3 (1.2%)	1 (2.0%)	2 (1.0%)		
**GENDER**	248				**0.009**	0.056
MALE		220 (89%)	40 (78%)	180 (91%)		
FEMALE		28 (11%)	11 (22%)	17 (8.6%)		
**AGE_AT_DIAGNOSIS**	248				0.091	0.4
<70		181 (73%)	42 (82%)	139 (71%)		

Variable	N	Overall, N = 248[1]	LKB1 LOSS, N = 51[1]	LKB1 INTACT, N = 197[1]	p-value[2]	q-value[3]
>=70		67 (27%)	9 (18%)	58 (29%)		
**pSTAGE**	248				0.2	0.5
IA		12 (4.8%)	3 (5.9%)	9 (4.6%)		
IB		25 (10%)	3 (5.9%)	22 (11%)		
IIA		51 (21%)	8 (16%)	43 (22%)		
IIB		37 (15%)	12 (24%)	25 (13%)		
IIIA		121 (49%)	24 (47%)	97 (49%)		
IIIB		1 (0.4%)	0 (0%)	1 (0.5%)		
IV		1 (0.4%)	1 (2.0%)	0 (0%)		
**LUACs_Micropapillary Component**	120				0.2	0.5
0%		95 (79%)	35 (85%)	60 (76%)		
<=5%		7 (5.8%)	2 (4.9%)	5 (6.3%)		
>5%		1 (0.8%)	1 (2.4%)	0 (0%)		
>=10%		17 (14%)	3 (7.3%)	14 (18%)		
**LUACs_Secondary Histological Pattern**	120				0.3	0.5

Variable	N	Overall, N = 248[1]	LKB1 LOSS, N = 51[1]	LKB1 INTACT, N = 197[1]	p-value[2]	q-value[3]
NO SECONDARY		42 (35%)	19 (46%)	23 (29%)		
LEPIDIC		6 (5.0%)	2 (4.9%)	4 (5.1%)		
ACINAR		31 (26%)	10 (24%)	21 (27%)		
PAPILLARY		17 (14%)	3 (7.3%)	14 (18%)		
MICROPAPILLARY		15 (13%)	3 (7.3%)	12 (15%)		
SOLID		9 (7.5%)	4 (9.8%)	5 (6.3%)		
**GRADE**	248				0.3	0.5
G1		14 (5.6%)	4 (7.8%)	10 (5.1%)		
G2		83 (33%)	13 (25%)	70 (36%)		
G3		151 (61%)	34 (67%)	117 (59%)		
**META STATUS**	248				0.4	0.6
LN META-		60 (24%)	10 (20%)	50 (25%)		
LN META+		188 (76%)	41 (80%)	147 (75%)		
**LUACs_Predominant Histological Pattern**	120				0.5	0.7
LEPIDIC		5 (4.2%)	1 (2.4%)	4 (5.1%)		
ACINAR		30 (25%)	8 (20%)	22 (28%)		

Variable	N	Overall, N = 248[1]	LKB1 LOSS, N = 51[1]	LKB1 INTACT, N = 197[1]	p-value[2]	q-value[3]
PAPILLARY		12 (10%)	4 (9.8%)	8 (10%)		
MICROPAPILLARY		1 (0.8%)	0 (0%)	1 (1.3%)		
SOLID		62 (52%)	27 (66%)	35 (44%)		
INVASIVE MUCINOUS		3 (2.5%)	0 (0%)	3 (3.8%)		
COLLOID		3 (2.5%)	0 (0%)	3 (3.8%)		
ENTERIC		4 (3.3%)	1 (2.4%)	3 (3.8%)		
**LN_STATUS**	248				0.6	0.7
LN0		60 (24%)	10 (20%)	50 (25%)		
LN1		106 (43%)	24 (47%)	82 (42%)		
LN2		21 (8.5%)	3 (5.9%)	18 (9.1%)		
LN3		2 (0.8%)	1 (2.0%)	1 (0.5%)		
LN1 & LN2		59 (24%)	13 (25%)	46 (23%)		
**Tumor_Size**	248				0.6	0.7
<=3cm		75 (30%)	13 (25%)	62 (31%)		
>3cm & <=5cm		79 (32%)	16 (31%)	63 (32%)		
5cm & <=7cm		48 (19%)	13 (25%)	35 (18%)		

Variable	N	**Overall**, N = 248[1]	**LKB1 LOSS**, N = 51[1]	**LKB1 INTACT**, N = 197[1]	p-value[2]	q-value[3]
>7cm		46 (19%)	9 (18%)	37 (19%)		
**pSTAGE_binary**	248				>0.9	>0.9
IIIa - IV		123 (50%)	25 (49%)	98 (50%)		
I & II		125 (50%)	26 (51%)	99 (50%)		

[1]n (%)

[2]Pearson's Chi-squared test; Fisher's exact test

[3]False discovery rate correction for multiple testing
